# Splicing factors control *C. elegans* behavioural learning in a single neuron by producing DAF-2c receptor

**DOI:** 10.1038/ncomms11645

**Published:** 2016-05-20

**Authors:** Masahiro Tomioka, Yasuki Naito, Hidehito Kuroyanagi, Yuichi Iino

**Affiliations:** 1 Molecular Genetics Research Laboratory, Graduate School of Science, The University of Tokyo, Bunkyo-ku, Tokyo 113-0033, Japan; 2 Department of Biological Sciences, Graduate School of Science, The University of Tokyo, Bunkyo-ku, Tokyo 113-0033, Japan; 3 Laboratory of Gene Expression, Medical Research Institute, Tokyo Medical and Dental University, Bunkyo-ku, Tokyo 113-8510, Japan

## Abstract

Alternative splicing generates protein diversity essential for neuronal properties. However, the precise mechanisms underlying this process and its relevance to physiological and behavioural functions are poorly understood. To address these issues, we focused on a cassette exon of the *Caenorhabditis elegans* insulin receptor gene *daf-2*, whose proper variant expression in the taste receptor neuron ASER is critical for taste-avoidance learning. We show that inclusion of *daf-2* exon 11.5 is restricted to specific neuron types, including ASER, and is controlled by a combinatorial action of evolutionarily conserved alternative splicing factors, RBFOX, CELF and PTB families of proteins. Mutations of these factors cause a learning defect, and this defect is relieved by DAF-2c (exon 11.5+) isoform expression only in a single neuron ASER. Our results provide evidence that alternative splicing regulation of a single critical gene in a single critical neuron is essential for learning ability in an organism.

## Abstract (web summary)

Little is known about the molecular mechanisms regulating neuron-specific alternative splicing. Here, the authors identify a combination of RNA-binding proteins regulating neuron-specific expression of the *C. elegans* insulin receptor isoform DAF-2c and find disrupting these factors leads to learning deficits.

## Graphical abstract

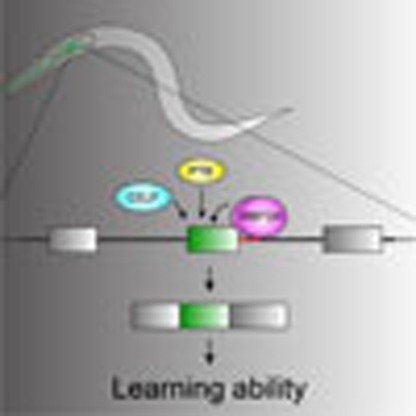

Diverse neuronal populations are essential for physiological functions of the brain. It is widely accepted that neuronal identities are determined by combinatorial actions of transcription factors that are controlled by regulatory networks of microRNAs and signal transduction pathways^1^,^2^. It has also been suggested that post-transcriptional regulation by alternative splicing plays critical roles in many steps of neuronal development^3^,^4^. Dysregulation of alternative splicing events in the brain has been linked to several neurodegenerative and neuropsychiatric disorders, highlighting the physiological importance of alternative splicing in the brain^5^,^6^. Several brain-enriched RNA-binding proteins (RBPs), such as NOVA, RBFOX and polypyrimidine tract-binding (PTB) families of proteins, have been found to regulate alternative splicing events in the nervous system^4^. Altered expression of these splicing factors causes substantial defects in survival and/or function of neurons^7^,^8^,^9^,^10^. Extensive studies using genome-wide approaches and computational methods have unveiled the neural splicing networks controlled by these proteins and some biologically relevant targets in brain function^5^,^7^,^11^,^12^. However, functional roles of such a considerable number of splicing isoforms in the brain are yet poorly understood.

Cell-type specificity of alternative splicing patterns can be ensured by combinatorial actions of alternative splicing factors^13^,^14^. Bioinformatic analysis using Bayesian networks reveals that *cis*-regulatory elements of neural splicing factors, NOVA and RBFOX, coexist in a number of sequences surrounding cassette exons, which implies their combinatorial splicing regulations of a set of neuronal genes^15^. Recent findings suggest that this type of regulation is used to determine neuronal subtype identities *in vivo*^16^. Combinatorial regulation of the CELF family protein, UNC-75, and the Hu/ELAV family protein, EXC-7, is required for alternative splicing of a JIP3 homologue *unc-16* in *Caenorhabditis elegans*. Predominant inclusion of a cassette exon of *unc-16* occurs in cholinergic neurons that co-express UNC-75 and EXC-7, but not GABAergic neurons that express only UNC-75, suggesting that predominant inclusion of the cassette exon is achieved by combinatorial expression of a pair of splicing factors^16^. The *in vivo* mechanism to generate diverse neuronal properties via alternative splicing is just beginning to be understood.

To analyse alternative splicing events and their relevance to physiological and behavioural functions, the *C. elegans* nervous system is useful. *C. elegans* hermaphrodites have only 302 neurons, which are classified into 118 classes based on their morphology and neural connectivity^17^. Among 60 sensory neurons, amphid sensory neurons comprising 12 neuron classes are the major sensors of environmental chemicals and temperature. *C. elegans* uses the amphid sensory neurons for innate and learned behavioural responses to seek suitable conditions and to avoid unfavourable conditions^18^. We have reported that insulin/IGF receptor signalling in the taste receptor neuron ASE right (ASER), the right-sided ASE class of amphid sensory neurons, plays a pivotal role in taste-avoidance learning during which worms learn to avoid salt concentrations encountered under starvation conditions^19^,^20^*. C. elegans* has two isoforms of the insulin/IGF receptor homologue, DAF-2a and DAF-2c, which are produced by skipping and inclusion of exon 11.5, respectively. Only DAF-2c (exon 11.5+) is preferentially transported to the axonal processes of the ASER neuron by interacting with a kinesin-1 motor complex. Strikingly, translocation of DAF-2c, but not DAF-2a (exon 11.5−), increases after food deprivation and can support taste-avoidance learning. In contrast, DAF-2a is more effective than DAF-2c in other *daf-2*-regulated processes, including dauer formation and longevity. Thus, DAF-2 isoforms play distinct roles in the biological processes, and DAF-2c has a critical role in the ASER neuron in taste-avoidance learning^20^.

Here we report that alternative splicing of *daf-2* exon 11.5 is regulated in a cell-type-specific manner. Using fluorescence-based splicing reporters and a messenger RNA (mRNA)-tagging method, we demonstrate that *daf-2* exon 11.5 inclusion occurs in restricted neuron classes, including ASE. We investigate the molecular mechanism that underlies this neuron-class-specific alternative splicing event. We find that *daf-2* exon 11.5 inclusion is regulated by RBFOX, CELF and PTB families of proteins, whose mammalian homologues act in the brain as alternative splicing factors. Furthermore, our analysis reveals that these splicing factors are co-expressed only in the restricted neuron classes, where *daf-2* exon 11.5 inclusion predominantly occurs. Ectopic expression of PTB-1/PTB was sufficient to induce predominant inclusion of *daf-2* exon 11.5 in the nervous system, suggesting that the PTB-1 expression is a critical determinant of the neuron-class specificity of exon 11.5 inclusion. We also show that mutations of these factors cause defects in taste-avoidance learning. Remarkably, expression of DAF-2c (exon 11.5+) only in a single neuron ASER relieved the learning defect of the splicing factor mutant. Thus, combinatorial action of the splicing factors in ASER determines the neuronal property required for learning ability by controlling alternative splicing of *daf-2*.

## Results

### Cell-type-specific alternative splicing of *daf-2* exon 11.5

**Figure 1:**
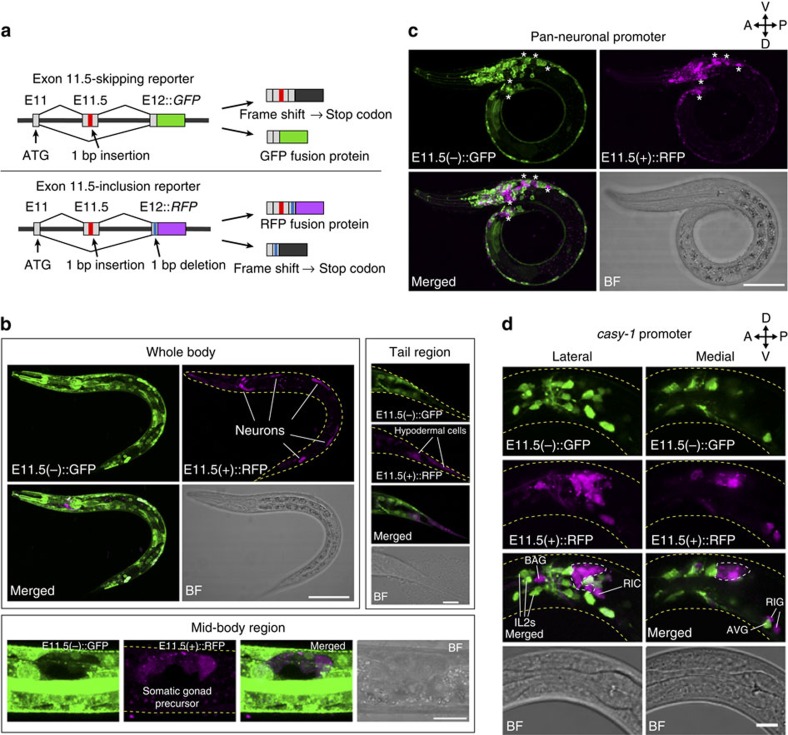
Cell-type-specific alternative splicing of *daf-2* exon 11.5. (**a**) Schematic of *daf-2* alternative splicing reporters for monitoring skipping (top) and inclusion (bottom) of exon 11.5. GFP and RFP fusion proteins are expressed by exon skipping and exon inclusion, respectively. (**b**–**d**) Expression patterns of the exon 11.5-skipping/inclusion reporters driven by a ubiquitous promoter, the *eef-1A.1* promoter (**b**), a pan-neuronal promoter, the *H20* promoter (**c**) and the *casy-1* promoter (**d**). Maximum intensity projection images of L1/L2 larvae. Asterisks in **c** represent neurons with predominant RFP expression. Lateral and medial positions of the head region of a worm carrying the neuronal exon 11.5-skipping/inclusion reporter (**d**). The predominant RFP signals are observed in the amphid sensory neurons (dotted region) as well as in other neuron classes, including BAG sensory neurons, RIC and RIG interneurons (**d**). The predominant GFP signals are observed in neurons, including IL2 sensory neurons and AVG interneurons (**d**). BF, bright field. The utilized worm strains were JN1737 (**b**), JN1736 (**c**) and JN785 (**d**), whose genotypes are shown in Supplementary Table 3. Scale bars, 50 μm (**b**, whole body); 30 μm (**c**); 10 μm (**b**, mid-body region and tail region, and **d**).

To visualize the alternative splicing patterns of *daf-2* exon 11.5, we created splicing reporters, in which either green fluorescent protein (GFP) or red fluorescent protein (RFP) complementary DNA (cDNA) was fused with the modified *daf-2* genomic fragments that contain exon 11.5, the flanking introns and parts of exons 11 and 12 to monitor exon skipping or exon inclusion, respectively (Fig. 1a; Supplementary Fig. 1a). When both reporters were expressed under a common ubiquitous promoter, GFP signals, which reflect exon skipping, were widely observed. In contrast, RFP signals, which reflect exon inclusion, were observed only in restricted cell types, including neurons, the somatic gonad and hypodermal cells in the tail region (Fig. 1b). When these reporters were exclusively expressed in the nervous system, GFP and RFP expressions were observed in distinct neurons in a mutually exclusive manner (Fig. 1c; Supplementary Movie 1), suggesting that exon 11.5 selection occurs in a neuron-class-specific manner.

To further investigate the reporter expression patterns in each neuron class, these reporters were expressed under several neuron-class-selective promoters. All the amphid sensory neurons, including ASER, predominantly expressed E11.5(+)::RFP (Fig. 1d; Supplementary Fig. 2a). The predominant RFP signals were also observed in some other neuron classes, including gas-sensing BAG and mechanosensory OLL neurons, and a small subset of inter- and motor-neurons (Fig. 1d; Supplementary Fig. 2b). By comparison, predominant GFP signals were observed in some other neuron classes, including IL2 sensory neurons, and inter- and motor-neurons (Fig. 1d; Supplementary Fig. 2b and c). Swapping of GFP and RFP cDNAs in the exon 11.5-skipping/inclusion reporters reversed the expression patterns of GFP and RFP (Supplementary Fig. 2d), which indicated that neuron-class-specific expression of the exon 11.5-skipping/inclusion reporters was not due to the different characteristics of these fluorescent proteins, but because of the differential splicing patterns.

**Figure 2:**
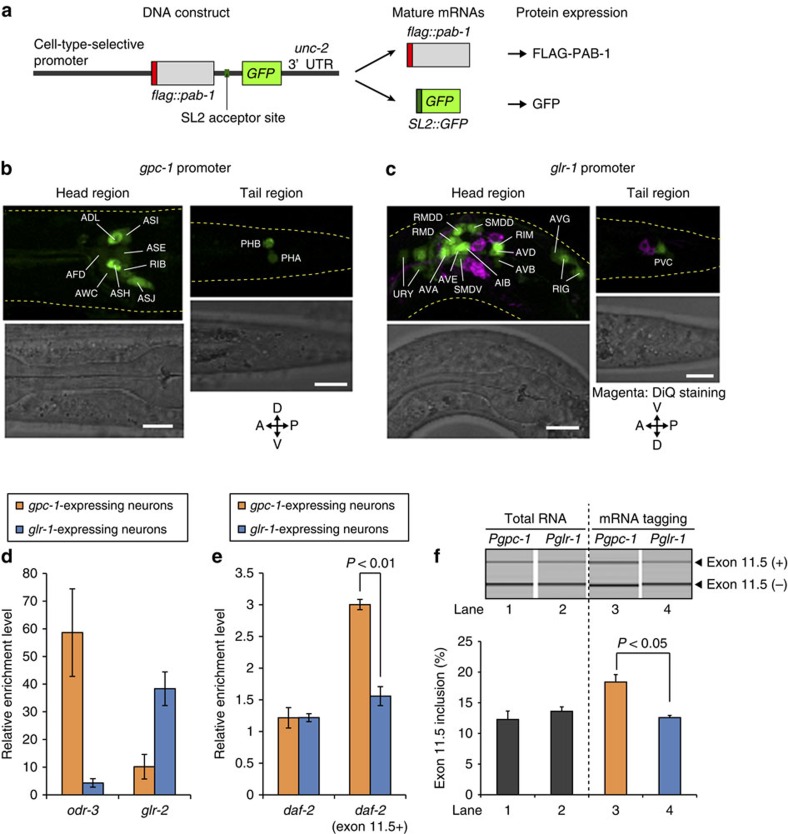
Neuron-class-specific exon 11.5 selection of endogenous *daf-2*. (**a**) Schematic of DNA constructs in transgenes used for cell-type-selective expression of FLAG-tagged poly(A)-binding protein (FLAG-PAB-1). An intergenic region between *flag::pab-1* and *GFP* contains an acceptor site of spliced leader (SL2) *trans*-splicing. The minigenes produce two mature mRNAs from a polycistronic pre-mRNA by *trans*-splicing, and GFP is co-expressed with FLAG-PAB-1 as a marker. (**b**,**c**) GFP expression patterns of a worm expressing the *flag::pab-1::SL2::GFP* minigene under the *gpc-1* (**b**) or the *glr-1* (**c**) promoter, respectively. The *gpc-1* promoter drives expression in 10 neuron classes, including 7 amphid sensory neurons (ADL, ASI, ASE, AFD, AWC, ASH and ASJ). The head and tail regions of L1 larvae are shown. Scale bars, 10 μm. (**d**,**e**) Enrichment levels of *odr-3* and *glr-2* mRNAs (**d**), and total *daf-2* and *daf-2* (exon 11.5+) mRNAs (**e**) relative to a ubiquitous gene, *eef-1A.1*, mRNA in *gpc-1*- and *glr-1*-expressing neurons. Relative mRNA levels were quantified by quantitative RT–PCR, and enrichment levels were calculated as ratios of the mRNA levels in neuron-type-specific poly(A)^+^ RNAs to those in total RNAs from whole worms. *odr-3* is exclusively expressed in some amphid sensory neurons^52^; *glr-2* is co-expressed with *glr-1* in many neurons^53^. (**f**) RT–PCR analysis of *daf-2* exon 11.5 using total RNAs from whole worms (lanes 1 and 2) and neuron-type-specific poly(A)^+^ RNAs (lanes 3 and 4), which were extracted from adult worms expressing FLAG-PAB-1 under the *gpc-1* promoter (lanes 1 and 3) or the *glr-1* promoter (lanes 2 and 4). The *y* axis represents molar ratio of exon 11.5(+) mRNA to the total. Error bars represent s.e.m. among experiments using three biological replicates, and *P* values were determined by two-tailed *t*-test (**d**–**f**). The utilized worm strains were JN1709 (**b**,**d**–**f**) and JN1710 (**c**,**d**–**f**), whose genotypes are shown in Supplementary Table 3.

To confirm neuron-class-specific alternative splicing of exon 11.5 in the endogenous *daf-2* transcripts, we isolated mRNAs specifically from the amphid sensory neurons or other classes of neurons, using an mRNA-tagging method^20^, and compared enrichment levels of total *daf-2* and the *daf-2* (exon 11.5+) isoform. We utilized the *gpc-1* and the *glr-1* promoters for expression of FLAG-tagged poly(A)-binding protein in 10 neuron classes, including 7 amphid sensory neurons, and in 15 classes of inter- and motor-neurons, respectively (Fig. 2a–d). Total *daf-2* mRNAs were enriched at comparable levels in the *gpc-1*- and *glr-1*-expressing neurons, whereas *daf-2* (exon 11.5+) mRNA was enriched at a significantly higher level in the *gpc-1*-expressing neurons (Fig. 2e). Reverse transcription–PCR (RT–PCR) analysis confirmed the significantly higher inclusion level of *daf-2* exon 11.5 in the *gpc-1*-expressing neurons than in the *glr-1*-expressing neurons (Fig. 2f). The expression patterns of the exon 11.5-skipping/inclusion reporters driven by these promoters (Supplementary Fig. 2a,c) were consistent with these results, confirming that the reporter expressions reflect alternative splicing patterns of the endogenous *daf-2* transcripts. Taken together with the previous finding that DAF-2c (exon 11.5+) expressed in the ASER amphid sensory neuron supports taste-avoidance learning^20^, these data are consistent with the idea that exon 11.5 inclusion predominantly occurs in neurons that require DAF-2c in a neuron-class-specific manner.

### Multiple RBPs regulate exon 11.5 selection

**Figure 3:**
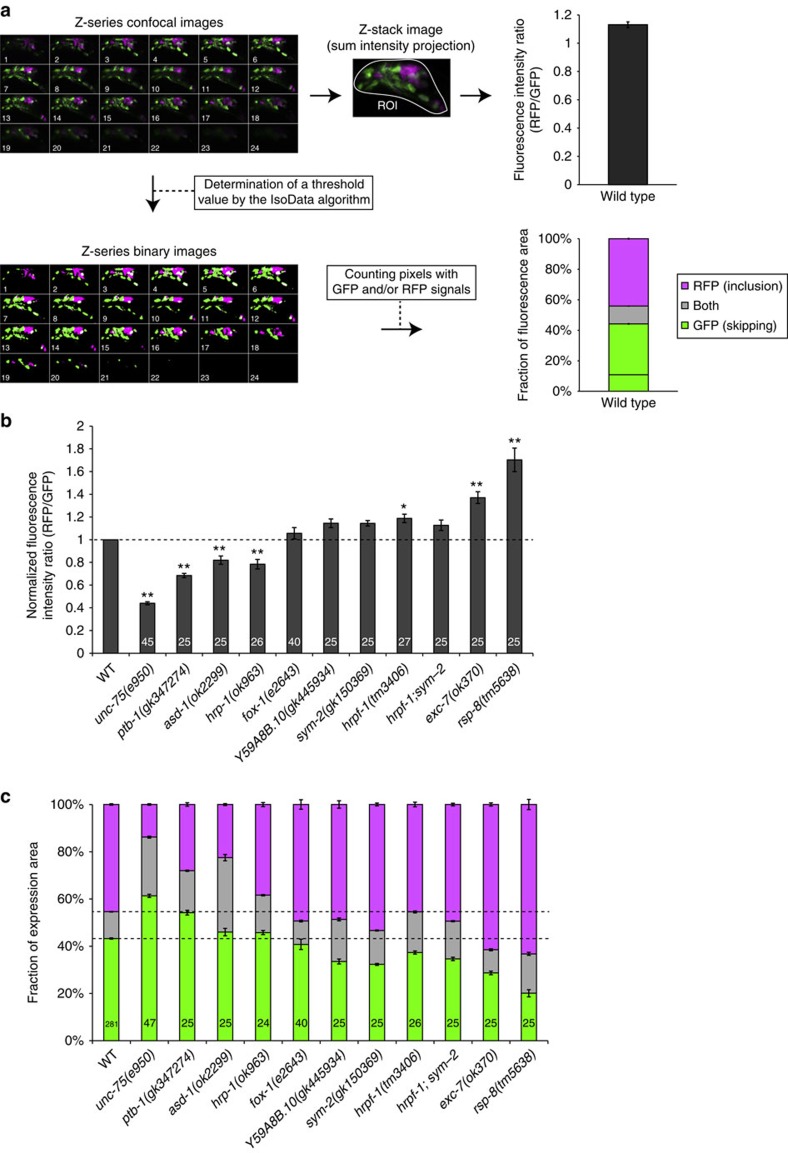
Multiple RBPs are required for exon 11.5 selection. (**a**) A flow chart for quantification of the neuronal exon 11.5-skipping/inclusion reporter expression. The top right graph shows the averaged RFP-to-GFP intensity ratio in the wild-type reporter worms with s.e.m. (*n*=514 worms). The bottom right graph shows the averaged fractions of GFP- and/or RFP-positive pixels in the wild-type reporter worms with s.e.m. (*n*=459 worms). See details in the Methods section. (**b**,**c**) Analysis of alternative splicing patterns of the neuronal exon 11.5-skipping/inclusion reporter in the mutant backgrounds of RBPs. Normalized fluorescence intensity ratios of RFP (exon inclusion) to GFP (exon skipping) (**b**) and fractions of GFP and RFP expression (**c**) in the head ganglia are shown. Data were normalized by the averaged values in the wild-type worms (*n*⩾25; **b**). The *n* values are shown in each bar. Error bars represent s.e.m. *P<0.05; **P<0.01, different from wild type, two-tailed *t*-test with Bonferroni correction.

To understand the mechanism underlying neuron-class-specific inclusion of *daf-2* exon 11.5, we investigated the effects of mutations of genes whose mammalian orthologues were known to encode alternative splicing factors in the brain^3^ (Supplementary Table 1). We compared the fluorescence signals between wild-type and mutant worms expressing the exon 11.5-skipping/inclusion reporters in a variety of neurons, including all the amphid sensory neurons and some inter- and motor-neurons, under the *casy-1* promoter^21^ (hereafter this transgene is referred to as ‘neuronal exon 11.5-skipping/inclusion reporter'; Fig. 1d). The fluorescence signals in the head ganglia were evaluated based on intensity ratios of RFP to GFP and fractions of GFP and RFP expression areas (Fig. 3a). Some of the mutants showed significant changes in the reporter expression compared with the wild-type worms. E11.5(+)::RFP (exon 11.5 inclusion) signals were significantly reduced in the mutants of a CELF family gene *unc-75*, a PTB protein orthologue *ptb-1*, an RBFOX family gene *asd-1* and an hnRNP A1 homologue *hrp-1* (Fig. 3b,c). On the contrary, E11.5(+)::RFP signals were increased in the mutants of an hnRNP F/H homologue *hrpf-1*, a Hu/ELAV family gene *exc-7* and a TRA2β orthologue *rsp-8* (Fig. 3b,c). These data indicated that a number of evolutionarily conserved alternative splicing factors were involved in precise control of alternative splicing of exon 11.5 in the nervous system. Except for a ubiquitously expressed *hrp-1* gene^22^, these genes were reported to be expressed in restricted cell types, raising the possibility that neuron-class-specific alternative splicing of exon 11.5 is ensured by a combinatorial action of the splicing factors. To explore this idea, we further investigated the roles of the RBFOX genes (*asd-1* and *fox-1*), *unc-75* and *ptb-1*, which are required for the inclusion of exon 11.5 in the nervous system.

### RBFOX proteins directly promote exon 11.5 inclusion

**Figure 4:**
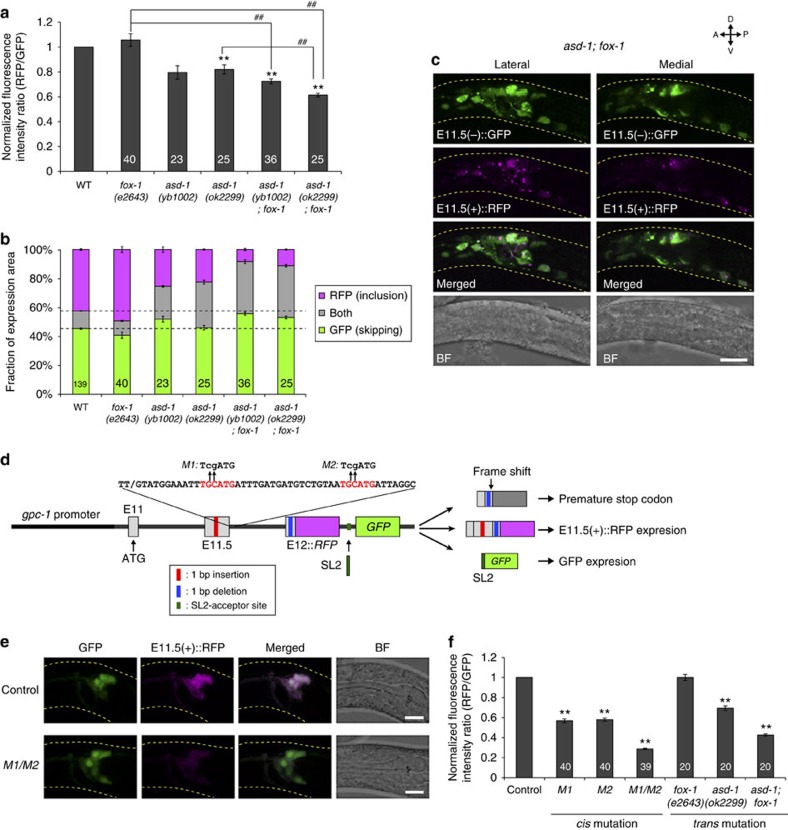
RBFOX family proteins directly promote exon 11.5 inclusion. (**a**,**b**) Normalized fluorescence intensity ratios of RFP to GFP (**a**) and fractions of GFP and RFP expression (**b**) of the neuronal exon 11.5-skipping/inclusion reporter in the mutants of the RBFOX family genes. Data were normalized by the averaged values in the wild-type worms (*n*⩾22, **a**). The same data as in Fig. 3b,c are presented for *fox-1(e2643)* and *asd-1(ok2299)*. (**c**) Maximum intensity projection images of the head region of a worm carrying the neuronal exon 11.5-skipping/inclusion reporter in the *asd-1(ok2299); fox-1(e2643)* background. (**d**) Schematic of modified *daf-2* exon 11.5-inclusion reporters. An intergenic region between RFP and GFP contains an acceptor site of SL2 *trans*-splicing, by which a polycistronic pre-mRNA is converted into monocistronic *daf-2* mini-gene-fused *RFP* mRNA and *GFP* mRNA. The RFP fusion protein is expressed upon exon 11.5 inclusion. Sites and sequences of the mutations *M1* and *M2* are indicated with lower case above the consensus RBFOX target stretches in red. (**e**) Expression patterns of the wild-type (control) and mutant form (*M1/M2*) of the modified exon 11.5-inclusion reporter driven by the *gpc-1* promoter. (**f**) Normalized fluorescence intensity ratios of RFP (exon inclusion) to GFP (promoter activity) in the amphid sensory neurons. Data were normalized by the averaged values in the wild-type worms (*n*⩾20). The *n* values are shown in each bar (**a**,**b**,**f**). Error bars represent s.e.m. **P<0.01, different from wild-type (**a**) and control (**f**) worms; ^##^*P*<0.01, different from each other, two-tailed *t*-test with Bonferroni correction. L1/L2 larvae were used for the analyses. Scale bars, 10 μm.

Exon inclusion of the neuronal exon 11.5-skipping/inclusion reporter was reduced in two *asd-1* mutants (Fig. 4a,b). A mutation in the paralogous gene *fox-1* enhanced the inclusion defect of *asd-1* mutants, and neurons with predominant exon 11.5 inclusion were severely reduced in the *asd-1; fox-1* double mutants (Fig. 4a–c; Supplementary Fig. 3a,b). Together with their common property as the RBFOX family splicing factor^23^, these data suggest functional redundancy of these genes for exon 11.5 inclusion. These RBFOX family proteins have been shown to regulate splicing through an evolutionarily conserved binding motif UGCAUG^23^,^24^. We found that the flanking intron downstream of *daf-2* exon 11.5 contains two putative RBFOX target stretches (Supplementary Fig. 1a). To test the involvement of these stretches in exon 11.5 inclusion, we created modified versions of the E11.5(+)::RFP reporter to monitor exon-inclusion levels relative to the reporter expression levels (Fig. 4d). Mutations in either of the UGCAUG stretches caused a significant reduction, and a double mutation of these stretches caused a strong reduction in the exon 11.5 inclusion, as did the *asd-1; fox-1* mutation (Fig. 4e,f). These data suggest that the RBFOX family proteins, ASD-1 and FOX-1, directly promote inclusion of *daf-2* exon 11.5 in the nervous system.

### Cooperative action of RBPs in exon 11.5 inclusion

**Figure 5:**
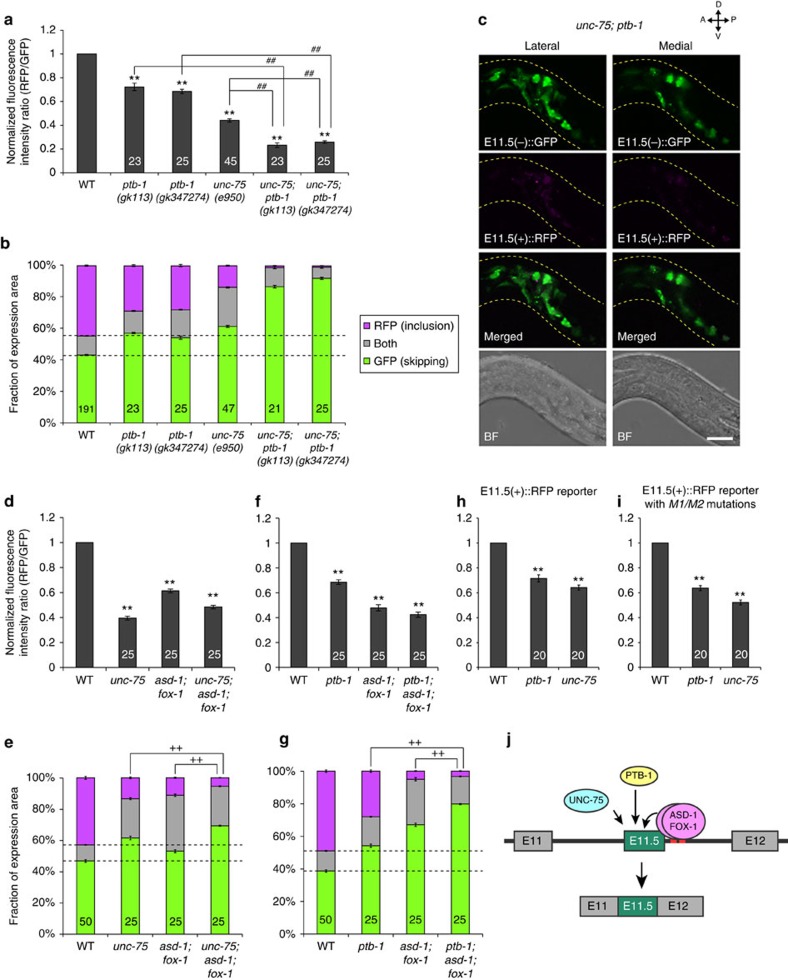
UNC-75, PTB-1 and the RBFOX proteins cooperatively regulate exon 11.5 inclusion. (**a**,**b**) Normalized fluorescence intensity ratios of RFP to GFP (**a**) and fractions of RFP and GFP expression (**b**) of the neuronal exon 11.5-skipping/inclusion reporter in the mutants. (**c**) Maximum intensity projection images of the head region of a worm carrying the neuronal exon 11.5-skipping/inclusion reporter in the *unc-75(e950); ptb-1(gk347274)* background. Scale bar, 10 μm. (**d**–**g**) Genetic interactions of the RBFOX mutant, *asd-1(ok2299); fox-1(e2643)*, with *unc-75(e950)* (**d**,**e**) and with *ptb-1(gk347274)* (**f**,**g**) in *daf-2* exon 11.5 splicing. Normalized fluorescence intensity ratios of RFP to GFP (**d**,**f**) and fractions of RFP and GFP expression (**e**,**g**) in the head ganglia of worms carrying the neuronal exon 11.5-skipping/inclusion reporter in the mutant backgrounds are shown. (**h**,**i**) Normalized fluorescence intensity ratios of RFP (exon inclusion) to GFP (promoter activity) of the modified *daf-2* exon 11.5-inclusion reporters with (**i**) or without (**h**) the *M1/M2* mutations in the wild-type, *unc-75(e950)* and *ptb-1(gk347274)* backgrounds. The reporter minigenes were expressed under the *gpc-1* promoter. (**j**) A regulatory model of *daf-2* exon 11.5 inclusion by a combinatorial action of RBFOX, CELF and PTB families of proteins. Data were normalized by the averaged values in the wild-type worms (*n*⩾24, **a**; *n*=25, **d** and **f**; *n*=20, **h** and **i**). The *n* values are shown in each bar. Error bars represent s.e.m. **P<0.01, different from wild type; ^##^*P*<0.01, different from each other; ^++^*P*<0.01, comparison of RFP-positive fractions, two-tailed *t*-test with Bonferroni correction. L1/L2 larvae were used for the analyses.

Exon inclusion of the neuronal exon 11.5-skipping/inclusion reporter was reduced in all seven *unc-75* mutants and two *ptb-1* mutants (Fig. 5a,b; Supplementary Fig. 4a,b). The *ptb-1* mutations enhanced the inclusion defect of a null mutant *unc-75(e950)* (Fig. 5a–c), suggesting that *unc-75* and *ptb-1* cooperatively regulate predominant inclusion of *daf-2* exon 11.5. We next tested for genetic interactions of *unc-75* and *ptb-1* with the RBFOX family genes (Fig. 5d–g). Each of the *unc-75* and *ptb-1* mutations slightly but significantly enhanced the reduced exon inclusion of the *asd-1; fox-1* double null mutant (Figs 4c and 5d–g; Supplementary Fig. 5). Furthermore, these mutations reduced exon inclusion of the modified E11.5(+)::RFP reporters (Fig. 4d) with or without the mutations in the RBFOX-binding stretches (Fig. 5h,i), which indicated that *unc-75* and *ptb-1* promoted exon 11.5 inclusion independently of the RBFOX actions. Collectively, a series of genetic analyses suggest that cooperative action of the three families of proteins, RBFOX family, UNC-75/CELF and PTB-1/PTB regulates predominant inclusion of *daf-2* exon 11.5 (Fig. 5j).

### Abnormal splicing of endogenous *daf-2* in RBP mutants

**Figure 6:**
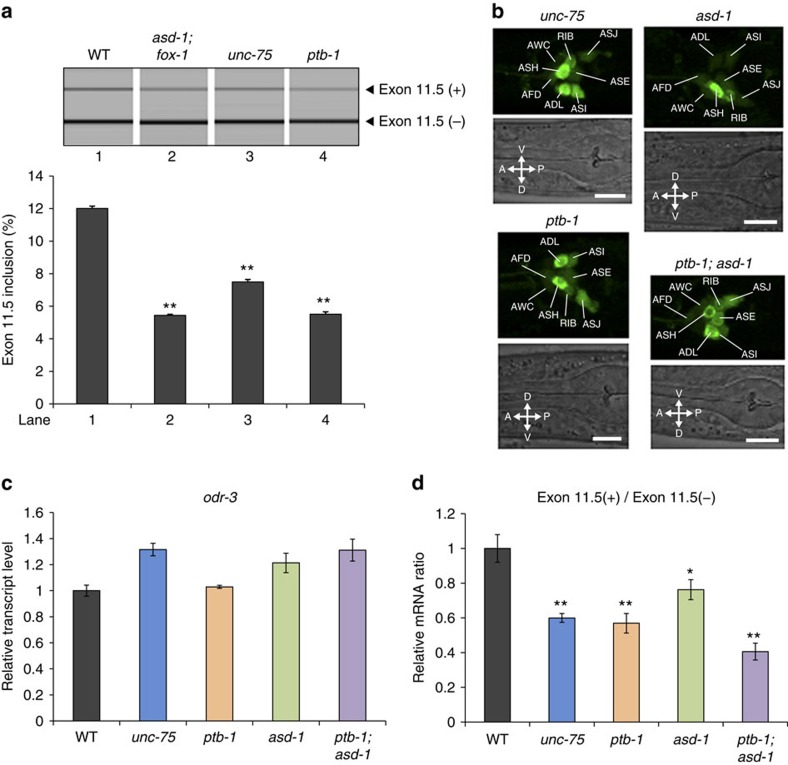
UNC-75, PTB-1 and RBFOX proteins promote exon 11.5 inclusion of endogenous *daf-2*. (**a**) RT–PCR analysis of *daf-2* exon 11.5 using total RNAs from the synchronized L1 larvae of wild-type, *asd-1(yb978); fox-1(e2643)*, *unc-75(yb1701)* and *ptb-1(gk347274)*. (**b**) GFP expression patterns of worms expressing the *flag::pab-1::SL2::GFP* minigene under the *gpc-1* promoter in *unc-75(e950)*, *ptb-1(gk347274)*, *asd-1(ok2299)* and *ptb-1(gk347274); asd-1(ok2299)* backgrounds. Note that expression of GFP is not affected in these mutants. The head regions of L1/L2 larvae are shown. Scale bars, 10 μm. (**c**,**d**) Relative transcript levels of *odr-3* (**c**) and relative levels of *daf-2* (exon 11.5+) to *daf-2* (exon 11.5−) mRNAs (**d**) in the *gpc-1*-expressing neurons, as determined by quantitative RT–PCR. Neuron-type-specific poly(A)^+^ RNAs were isolated from adult worms expressing FLAG-PAB-1 under the *gpc-1* promoter in the wild-type, *unc-75(e950)*, *ptb-1(gk347274)*, *asd-1(ok2299)* and *ptb-1(gk347274); asd-1(ok2299)* backgrounds. The same RNA samples were used in **c** and **d**. Data were normalized by the averaged values in the wild-type worms. Error bars represent s.e.m. among three technical replicates. *P<0.05; **P<0.01, different from wild type, one-way analysis of variance (ANOVA) followed by Dunnett *post hoc* test.

We next examined requirements of UNC-75, PTB-1 and the RBFOX proteins for neuron-class-specific alternative splicing of endogenous *daf-2*. RT–PCR analysis using total RNAs from whole worms revealed that inclusion of *daf-2* exon 11.5 was significantly reduced in the splicing factor mutants compared with the wild type (Fig. 6a). We then analysed *daf-2* isoform expression in the amphid sensory neurons by the mRNA-tagging method. We used RNA fractions comparably enriched for transcripts in the amphid sensory neurons from the wild-type and the mutant worms for the analyses (Fig. 6b,c). Amount of the *daf-2* (exon 11.5+) isoform relative to the *daf-2* (exon 11.5−) isoform was significantly reduced in the amphid sensory neurons of the *unc-75*, *ptb-1* and *asd-1* single mutants, and a *ptb-1; asd-1* double mutant (Fig. 6d). These results confirmed that these splicing factors promote inclusion of exon 11.5 of endogenous *daf-2* in the amphid sensory neurons.

### The RBFOX genes are expressed in various neuron classes

**Figure 7:**
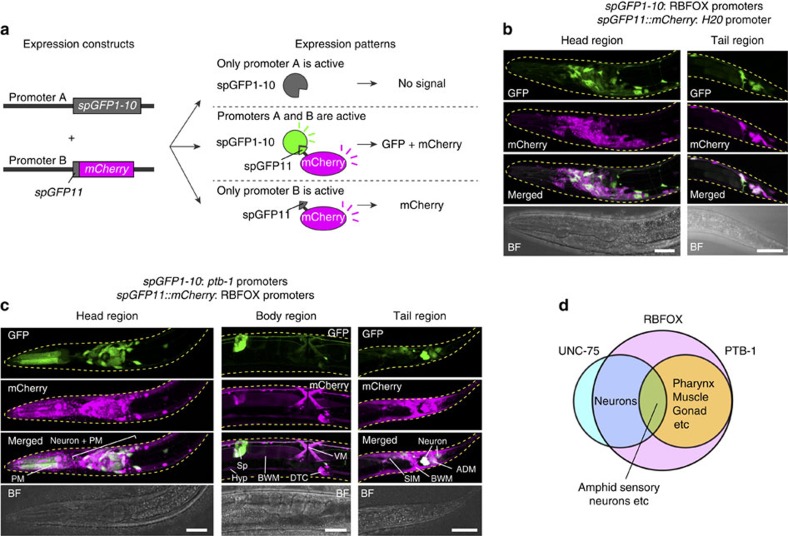
Split-GFP reporters reveal cell types that co-express *unc-75*, *ptb-1* and RBFOX genes. (**a**) Schematic of split-GFP reporters. When *split-GFP* fragments (*spGFP1-10* and *spGFP11::mCherry*) are expressed by distinct promoters, GFP signals are observed in the co-expression sites of the two *split-GFP* fragments. (**b**,**c**) Maximum intensity projection images of worms expressing spGFP1-10 and spGFP11::mCherry by the RBFOX (*asd-1* and *fox-1*) promoters and *H20* promoter, respectively (**b**), or the *ptb-1* (*ptb-1a* and *ptb-1b*) promoters and RBFOX (*asd-1* and *fox-1*) promoters, respectively (**c**). The head and tail regions of L2 larvae (**b**,**c**) and the body region of an adult worm (**c**) are shown. Scale bars, 20 μm (**b**,**c**, head and tail region); 40 μm (**c**, body region). ADM, anal depressor muscle; BWM, body wall muscle; DTC, distal tip cell; Hyp, hypodermis; PM, pharyngeal muscle; Sp, spermatheca; SIM, stomatointestinal muscle; VM, vulval muscle. (**d**) Summary of expression sites of *unc-75*, *ptb-1* and the RBFOX genes. They are co-expressed in a small subset of neurons, including amphid sensory neurons.

To test the hypothesis that neuron-class-specific inclusion of exon 11.5 is achieved by combinatorial expression of the splicing factors, we examined expression patterns of the RBFOX genes (*asd-1* and *fox-1*), *unc-75* and *ptb-1*. As previously reported^25^, the RBFOX genes were expressed in various tissues including the pharynx, the intestine, hypodermis, neurons, muscles and the somatic gonad (Supplementary Fig. 6a–g). As the neuronal *asd-1* expression was difficult to observe because of its expression in various tissues in the head region, we utilized split GFP^26^ to observe neuronal expression pattern of the RBFOX genes (Fig. 7a). One of the two split-GFP fragments, spGFP1-10, which contains 10 of the 11 strands of the β-barrel structure of GFP, was expressed under the *asd-1* and the *fox-1* promoters. mCherry-fused spGFP11, another split-GFP fragment that contains the 11th strand of the GFP β-barrel, was expressed by the pan-neuronal *H20* promoter. GFP signals, which reflect co-expression of spGFP1-10 and spGFP11::mCherry (Fig. 7a), were observed in many but not all neuron classes, suggesting that the expression of the RBFOX genes covers a variety of neuron classes (Fig. 7b).

### *ptb-1* is expressed in subsets of tissues

**Figure 8:**
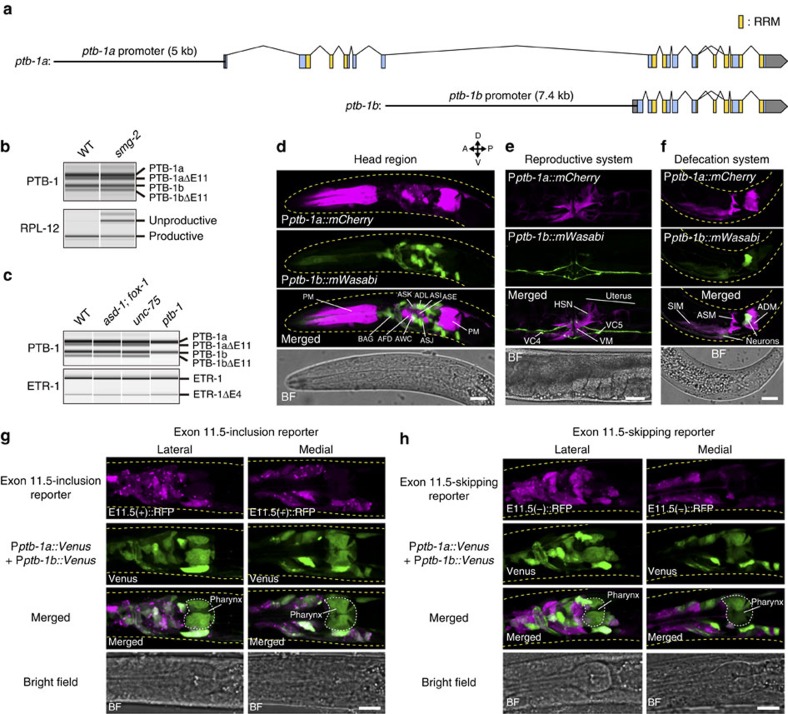
Inclusion of exon 11.5 predominantly occurs in *ptb-1*-expressing neurons. (**a**) Schematic representation of *ptb-1* isoforms. The upstream regulatory regions used in the transcriptional reporters are shown (black lines). Boxes indicate exons. Blue and grey areas represent coding and untranslated regions, respectively. Yellow areas represent RRM-encoding regions. (**b**) RT–PCR analysis of PTB-1 transcripts in synchronized L1 larvae of the wild-type (WT) and the *smg-2(yb979)* mutant. Percent-spliced-in (PSI) values of the PTB-1a isoforms are 77.6 and 65.2 for WT and *smg-2*, respectively, and those of the PTB-1b isoforms are 77.4 and 64.6, respectively. Stabilization of unproductive RPL-12 transcript confirms NMD deficiency of the *smg-2* mutant. (**c**) RT–PCR analysis of PTB-1 transcripts in synchronized L1 larvae of the wild-type and *asd-1(yb978); fox-1(e2643)*, *unc-75(yb1701)* and *ptb-1 (gk347274)* mutants. Note that the splicing pattern of *etr-1*, encoding an ELAV-type RBP, is not affected in any of the mutants. (**d**–**f**) Expression patterns of P*ptb-1a::mCherry* and P*ptb-1b::mWasabi*. Maximum intensity projection images of the head (**d**) and tail (**f**) regions of L1/L2 larvae and the mid-body region of an adult (**e**) are shown. ADM, anal depressor muscle; ASM, anal sphincter muscle; PM, pharyngeal muscle; SIM, stomatointestinal muscle; VM, vulval muscle. (**g**,**h**) Expression patterns of P*ptb-1a::Venus* plus P*ptb-1b::Venus* in combination with that of the exon 11.5-inclusion (**g**) or -skipping (**h**) reporter driven by the *H20* promoter. Maximum intensity projection images of the head region of L1/L2 larvae are shown. Scale bars, 10 μm (**d**,**f**–**h**); 30 μm (**e**).

Large-scale expression analyses suggested that *ptb-1* was expressed in a subset of neurons as well as non-neuronal cells^27^,^28^. We now precisely determined the expression pattern of *ptb-1*. We first confirmed expression of two *ptb-1* variants, *ptb-1a* and *ptb-1b*, driven by alternative promoters (Fig. 8a), as annotated in WormBase (http://www.wormbase.org). PTB-1a has four evolutionarily conserved RNA recognition motifs (RRM1−4)^29^, while PTB-1b lacks the first RRM (RRM1; Fig. 8a).

We also found a 34-nucleotide (nt) cassette exon, exon 11 (Fig. 8a,b), skipping of which causes a frameshift and a premature termination codon. RT–PCR analysis of the PTB-1 isoforms in the nonsense-mediated mRNA decay (NMD)-deficient *smg-2* mutant indicated that both *ptb-1a* and *ptb-1b* have the exon 11(−) isoforms destabilized by NMD (Fig. 8b). The exon 11(−) *ptb-1*variants were not detected in the *ptb-1* mutant (Fig. 8c), indicating that PTB-1 proteins repress splicing of exon 11 in their own transcripts to produce unproductive mRNA isoforms. The overall domain structure of PTB-1a and the mechanism of negative autoregulation of its own expression level via alternative splicing of the 34-nt cassette exon encoding a portion of the third RRM coupled with the NMD pathway are quite similar to those of its mammalian orthologues PTBP1 and PTBP2 (refs 30, 31), suggesting their conserved functions as alternative splicing regulators.

We next investigated the expression patterns of transcriptional reporter genes containing the upstream regulatory elements for either of the first exons (Fig. 8a). The *ptb-1a* promoter drove the expression in a small subset of neurons, including all the amphid sensory neurons, muscles and somatic gonadal cells, while the *ptb-1b* promoter drove strong expression in neurons (Fig. 8d–f). In the nervous system, the *ptb-1a* and *ptb-1b* promoters drove the expression in distinct but partially overlapping neuron classes, including ASE (Fig. 8d).

### Neural PTB-1 expression correlates with exon 11.5 inclusion

We then examined cell types that express *ptb-1* along with the RBFOX genes using split GFP (Fig. 7a). spGFP1-10 and spGFP11::mCherry were expressed under the two *ptb-1* (*ptb-1a* and *ptb-1b*) promoters and the two RBFOX gene (*asd-1* and *fox-1*) promoters, respectively. GFP signals, which reflect co-expression of *ptb-1* and either of the RBFOX genes, were observed in subsets of neuronal, muscle and somatic gonadal cells (Fig. 7c). This expression pattern was highly similar to that of the *ptb-1* transcriptional reporter genes (Fig. 8d–f), suggesting that RBFOX was expressed in most *ptb-1*-expressing cell types. Pan-neuronal expression of *unc-75* (ref. 32) further restricted cell types that co-express the RBFOX, *unc-75* and *ptb-1* genes to a subset of neurons (Fig. 7d; Supplementary Fig. 6h).

A series of expression analyses raised the possibility that the *ptb-1*-expressing neurons (that is, co-expression sites of the RBFOX, *unc-75* and *ptb-1* genes) might correlate with predominant inclusion of *daf-2* exon 11.5, which requires combinatorial action of these splicing factors. Therefore, we compared expression patterns of the *ptb-1* transcriptional reporters with that of the *daf-2* exon 11.5-inclusion or -skipping reporter in the nervous system. All neurons that expressed *ptb-1a::Venus* and/or *ptb-1b::Venus* also expressed E11.5(+)::RFP (Fig. 8g). In contrast, expression of Venus and E11.5(−)::RFP was mutually exclusive in many neuron classes (Fig. 8h). When the E11.5(+)::RFP and E11.5(−)::GFP reporters were co-expressed under the *ptb-1a* or *ptb-1b* promoter, the nervous system preferentially expressed E11.5(+)::RFP, with a few exception such as in AVA and RID neurons (Supplementary Fig. 2e,f), which further confirmed preferential inclusion of exon 11.5 in the PTB-1-expressing neurons. These analyses revealed strong correlation between PTB-1 expression and inclusion of *daf-2* exon 11.5 in the nervous system.

### PTB-1 confers neuron-type specificity on exon 11.5 selection

**Figure 9:**
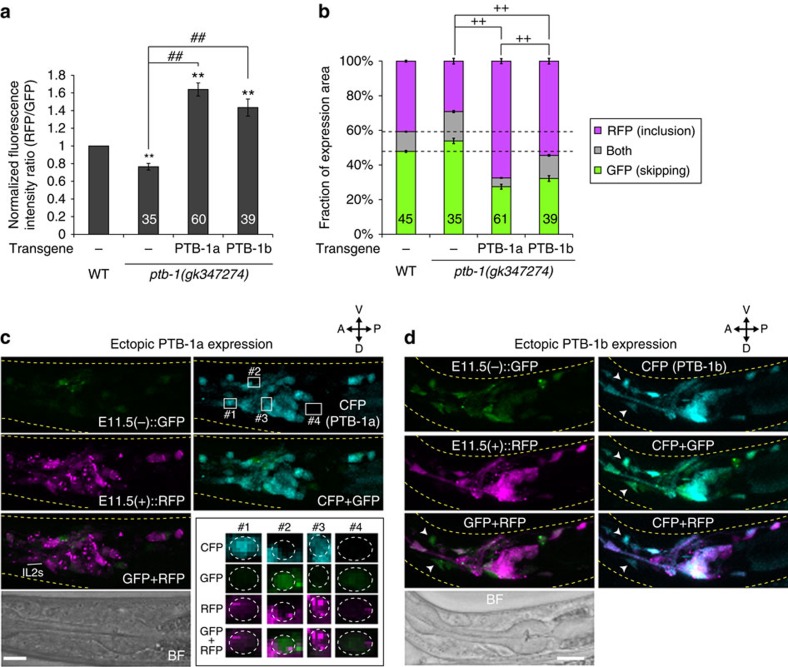
Ectopic PTB-1 expression promotes exon 11.5 inclusion in the nervous system. (**a**,**b**) Normalized fluorescence intensity ratios of RFP to GFP (**a**) and fractions of GFP and RFP expression (**b**) of the neuronal exon 11.5-skipping/inclusion reporter in the *ptb-1(gk347274)* mutant and in those expressing either PTB-1a or PTB-1b under the *casy-1* promoter. Data were normalized by the averaged values in the wild-type worms (*n*⩾33, **a**). The *n* values are shown in each bar. Error bars represent s.e.m. **P<0.01, different from wild type; ^##^*P*<0.01, different from each other; ^++^*P*<0.01, comparison of RFP-positive fractions, two-tailed *t*-test with Bonferroni correction. (**c**,**d**) Maximum intensity projection expression images of the neuronal exon 11.5-skipping/inclusion reporter in the *ptb-1(gk347274)* mutant expressing PTB-1a (**c**) or PTB-1b (**d**) under the *casy-1* promoter. CFP is co-expressed along with PTB-1 from a polycistronic pre-mRNA. As PTB-1 was expressed from extrachromosomal arrays (Supplementary Table 4), they exhibited mosaic expression patterns. High-magnification views of four regions depicted in the CFP image (**c**, top right) are shown in the bottom right inset in **c**. The areas surrounded by dotted lines indicate neuronal cell bodies with (#1 and 3) or without (#2 and 4) PTB-1a (CFP) expression (**c**). Arrowheads in **d** indicate neuronal cell bodies that express E11.5(−)::GFP, but not E11.5(+)::RFP, despite PTB-1b (CFP) expression. L1/L2 larvae were used for the analyses. Scale bars, 10 μm.

To test whether PTB-1 expression is sufficient for preferential inclusion of *daf-2* exon 11.5 in the nervous system, either PTB-1a or PTB-1b was ectopically expressed in a variety of neurons of the *ptb-1* mutant carrying the neuronal exon 11.5-skipping/inclusion reporter. Expression of either of the PTB-1 isoforms significantly enhanced exon 11.5 inclusion in the nervous system of the *ptb-1* mutant (Fig. 9a,b). The effect of ectopic PTB-1a expression was prominent compared with that of PTB-1b (Fig. 9b). Lack of the RRM1 domain in the PTB-1b isoform implies the requirement of RRM1 for efficient PTB-1 action. Most PTB-1a-expressing neurons, even the neurons where the E11.5(−)::GFP reporter was predominantly expressed in the wild type, such as IL2 sensory neurons (Fig. 1d), exhibited preferential expression of the E11.5(+)::RFP reporter, whereas neurons that failed to express PTB-1a did not (Fig. 9c). PTB-1b expression also caused preferential E11.5(+)::RFP expression in many neurons, although some neurons showed preferential E11.5(−)::GFP expression despite PTB-1b expression (Fig. 9d). These data indicated that the PTB-1 expression is a primary determinant of the neuron-class specificity of exon 11.5 inclusion in the nervous system.

### Impaired learning in RBP mutants

**Figure 10:**
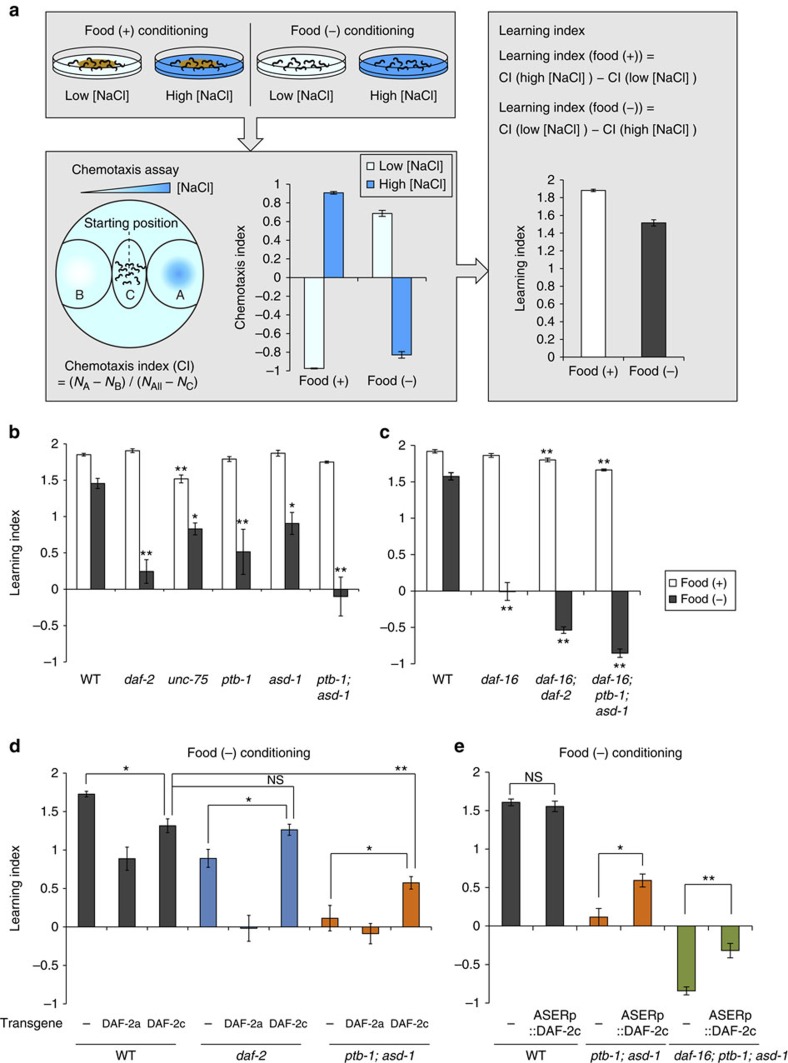
DAF-2c expression in ASER relieves impaired learning of the RBP mutant. (**a**) A flow chart of salt concentration learning assay. For conditioning, worms were exposed to low or high NaCl concentrations (low [NaCl] or high [NaCl], respectively) under fed or starvation conditions (food (+) conditioning or food (−) conditioning, respectively). After conditioning, chemotaxis to NaCl was tested on an agar plate with a NaCl gradient, and then chemotaxis and learning indices were calculated. See details in the Methods section. Chemotaxis and learning indices of wild-type worms are shown (*n*=21). (**b**) Salt concentration learning of the wild type, *daf-2(pe1230), unc-75(yb1714)*, *ptb-1(gk347274)*, *asd-1(ok2299)* and *ptb-1(gk347274); asd-1(ok2299)* mutants. (**c**) Salt concentration learning of the wild type, *daf-16(mgDf50)*, *daf-16(mgDf50); daf-2(e1370)* and *daf-16(mgDf50); ptb-1(gk347274); asd-1(ok2299)*. (**d**) Salt concentration learning of wild-type, *daf-2(pe1230)* and *ptb-1(gk347274); asd-1(ok2299)* worms expressing none (−) or either of the DAF-2 isoforms under the *H20* promoter. Salt conditioning was performed under starvation conditions. (**e**) Salt concentration learning of wild-type, *ptb-1(gk347274); asd-1(ok2299)* and *daf-16(mgDf50); ptb-1(gk347274); asd-1(ok2299)* worms expressing none (−) or DAF-2c only in ASER under the *gcy-5* promoter. Salt conditioning was performed under starvation conditions. *n*⩾4 assays (**b**,**c**). *n*⩾6 assays (**d**,**e**). Error bars represent s.e.m. *P<0.05; **P<0.01, different from wild-type or control worms, one-way analysis of variance (ANOVA) followed by Dunnett *post hoc* test (**b**–**d**) or two-tailed *t*-test with Bonferroni correction (**e**).

We explored the behavioural consequences of defective inclusion of *daf-2* exon 11.5 in the splicing factor mutants. *C. elegans* worms alter their preferences for NaCl concentrations after conditioning with different NaCl concentrations. They are attracted to NaCl concentrations at which they were fed, whereas they avoid NaCl concentrations at which they were starved (Fig. 10a)^33^. Insulin-like signalling mutants exhibit defects in salt concentration avoidance after starvation conditioning, which we call taste-avoidance learning^20^,^33^. As only the DAF-2c (exon 11.5+) isoform can mediate signalling for taste-avoidance learning^20^, we tested salt concentration learning of the splicing factor mutants.

As the *unc-75* null mutant exhibits a severe locomotion defect^24^, we examined reduction-of-function mutants of *unc-75.* The *unc-75(yb1714)* mutant showed defects in migration to higher salt concentrations (Supplementary Fig. 7), suggesting that *unc-75* is required for proper behavioural responses to salt as well as for locomotion. *ptb-1* and *asd-1* mutants had no obvious defect in behavioural responses to salt after conditioning under fed conditions, whereas they showed defects in taste-avoidance learning (Fig. 10b; Supplementary Fig. 8a,b). The *ptb-1; asd-1* double mutants showed further impaired taste-avoidance learning (Fig. 10b). These data indicated that *ptb-1* and *asd-1* are required for taste-avoidance learning.

To further characterize the behavioural phenotype of the splicing factor mutant, we tested for genetic interactions between the splicing factor genes and *daf-16*. DAF-2 signalling controls the action of the DAF-16/FOXO transcription factor, by inhibiting its nuclear translocation^34^. Most phenotypes of *daf-2* mutants are suppressed by *daf-16* mutations^34^, whereas we reported that *daf-16* mutations strongly enhanced the taste-avoidance learning defect of *daf-2*, implying that, unlike the canonical DAF-2 signalling, the insulin-like signalling mediated by DAF-2c (exon 11.5+) may regulate taste-avoidance learning in parallel with DAF-16 (ref. 19). *daf-16; ptb-1; asd-1* triple mutants showed largely normal behavioural responses to salt after conditioning under fed conditions, whereas they showed a strong defect in taste-avoidance learning as if they were not starved during conditioning (Fig. 10c; Supplementary Fig. 8c,d). This behavioural phenotype resembled that of the *daf-16; daf-2* mutant (Fig. 10c; Supplementary Fig. 8c,d)^19^. The similar genetic interactions of the *daf-16* gene with *ptb-1; asd-1* and *daf-2* support the idea that the splicing factors regulate taste-avoidance learning through the production of DAF-2c (exon 11.5+) isoform.

### DAF-2c expression in ASER relieves the impaired learning

To assess impacts of altered expression of *daf-2* isoforms on the taste-avoidance learning, we generated wild-type and mutant worms ectopically expressing either of the DAF-2 isoforms. Neuronal expression of either DAF-2a (exon 11.5−) or DAF-2c (exon 11.5+) in the wild-type resulted in impaired learning (Fig. 10d), suggesting that appropriate expression levels of DAF-2a and DAF-2c or their balance in the nervous system is important for proper learning ability. On the other hand, neuronal expression of DAF-2c, but not that of DAF-2a, rescued the learning defect of the *daf-2* mutant (Fig. 10d), which is consistent with the finding that only DAF-2c can support the taste-avoidance learning^20^. Similarly, neuronal expression of DAF-2c, but not of DAF-2a, partially restored the impaired learning of the *ptb-1; asd-1* mutant (Fig. 10d), indicating that the learning defect of the *ptb-1; asd-1* mutant is due at least in part to deceased expression of DAF-2c in the nervous system. Furthermore, DAF-2c expression only in the ASER neuron rescued the learning defect of the *ptb-1; asd-1* mutant (Fig. 10e), indicating that ASER is the critical neuron for the splicing factors to control taste-avoidance learning by producing DAF-2c. The DAF-2c expression in ASER also rescued the learning defect of the *daf-16; ptb-1; asd-1* mutant (Fig. 10e), suggesting that the DAF-2c (exon 11.5+) signalling regulates taste-avoidance learning in a DAF-16-independent manner. Collectively, these data demonstrate that the learning defect of the splicing factor mutant is mainly caused by the reduction of the DAF-2c (exon 11.5+) isoform in ASER.

## Discussion

In this study, we showed cell-type-specific alternative splicing of *daf-2* exon 11.5. Within the nervous system, *daf-2* exon 11.5 inclusion preferentially occurs in a restricted subset of neurons, including the amphid sensory neurons. This neuron-class-specific alternative splicing is determined by the combinatorial action of the evolutionarily conserved RBPs, RBFOX proteins (ASD-1 and FOX-1), UNC-75/CELF and PTB-1/PTB. We found that PTB-1, which is co-expressed with the RBFOX proteins and UNC-75 in the restricted neuron classes (Fig. 7d), has a key role in conferring neuron-class specificity of exon 11.5 inclusion. The combinatorial mutations of these splicing factors result in a pronounced defect in taste-avoidance learning. DAF-2c (exon 11.5+) expression only in a single neuron ASER relieves the learning defect of the splicing factor mutant. Therefore, the precisely regulated *daf-2* exon 11.5 inclusion in ASER is essential for taste-avoidance learning (Supplementary Fig. 9). DAF-2 signalling is also required for starvation-induced odour chemotaxis learning in AWC olfactory neurons^35^, although a DAF-2 isoform that regulates olfactory learning has not been determined. The predominant inclusion of exon 11.5 in the amphid sensory neurons, including AWC (Supplementary Fig. 2a), implies a requirement of the DAF-2c isoform in a range of sensory plasticity, including those of taste and smell responses.

PTB functions as both a repressor and an activator in many alternative splicing events in mammalian cells^31^,^36^. The neuronal PTB knockout mice show severe defects in neurogenesis and result in death soon after birth^8^,^12^. In this study, we unveiled the role of *C. elegans* PTB-1 in determination of the neuronal property essential for behavioural learning. The expression pattern and the ectopic expression analyses suggest that PTB-1 is a strong determinant of *daf-2* exon 11.5 inclusion in the nervous system. We characterized the two PTB-1 isoforms, PTB-1a and PTB-1b, which are produced by alternative promoters. PTB-1a strongly activates exon 11.5 inclusion compared with PTB-1b. Mammalian PTB proteins, PTBP1 and PTBP2, also have different effects on alternative splicing. It was reported that a switch between expressions of PTBP1 and its paralogue PTBP2 is required for reprogramming of protein isoforms during neuronal maturation^12^,^31^. This switch in expression is due in part to PTBP1-induced alternative splicing of a 34-nt cassette exon of PTBP2, which leads to mRNA degradation by NMD. We found the cassette exon (exon 11) in the *ptb-1* gene, skipping of which leads to NMD-dependent mRNA degradation. It is surprising that its role in negative autoregulation as well as its position and size is highly conserved between mammals and *C. elegans*. The two PTB-1 isoforms are expressed in distinct but partially overlapping neurons, including ASER. It would be interesting to investigate how PTB-1 isoform expressions are controlled at levels of transcription and post transcription to determine the neuronal properties essential for learning ability.

Except for *daf-2* exon 11.5 and *ptb-1* exon 11, we identified in this study, a target exon of PTB-1 has not been reported. The RBFOX proteins regulate several alternative splicing events in various tissues, including the muscles and the nervous system^23^,^24^. UNC-75 is exclusively expressed in the nervous system and determines protein isoforms of various neuronal genes^16^,^37^. Each of these splicing factors might control a different subset of neuronal genes. Because DAF-2c (exon 11.5+) expression only partially restores the taste-avoidance learning defect of the splicing factor mutant, other genes related to behavioural learning may be affected in the mutant. It would be interesting to identify exons cooperatively regulated by these splicing factors, which may be involved in learning ability. And this would provide further insights into how the neuron-class-specific alternative splicing contributes to the complex neuronal functions underlying learning.

*daf-2* exon 11.5 inclusion preferentially occurs also in some non-neuronal cells, including somatic gonadal cells. The DAF-2 signalling in the proximal region of somatic gonad, including uterine and spermathecal cells, regulates age-related loss of the germline stem cells^38^. Our results suggest that *ptb-1* and the RBFOX genes are co-expressed not only in neurons but also in uterine and spermathecal cells. These observations raise the intriguing possibility that the combinatorial action of PTB-1 and the RBFOX proteins might also regulate gonadal cell-type-specific inclusion of *daf-2* exon 11.5 and be related to the germline stem cell maintenance. It will be interesting to investigate the isoform-specific function of DAF-2 and requirement of cell-type-specific alternative splicing for maintenance of the germline stem cell number.

In addition to these splicing factors, we showed that mutations in *exc-7* and *rsp-8* increase expression of the neuronal exon 11.5-inclusion reporter. EXC-7/Hu/ELAV and RSP-8/TRA2β might repress the exon 11.5 inclusion and further fine-tune the neuron-type specificity of alternative splicing of *daf-2*. Further investigation is needed to clarify the functional role of the candidate repressors of exon 11.5 inclusion in the nervous system. On the other hand, the *hrp-1* mutation reduces expression of the neuronal exon 11.5-inclusion reporter. HRP-1/hnRNP A1 is ubiquitously expressed and essential for larval development^22^,^39^. RNA interference knockdown of *hrp-1* during the adult stage extends lifespan in a *daf-16*-dependent manner similar to that of *daf-2* (ref. 39). It will be interesting to determine whether and how HRP-1 contributes to selection of *daf-2* exon 11.5 and longevity extension.

We demonstrate that the RBFOX family proteins directly act through the binding motif UGCAUG in the intron downstream of exon 11.5. We identified a cassette exon homologous to *daf-2* exon 11.5 in *Cbr-daf-2*, the *daf-2* orthologue of the closely related nematode species *C. briggsae*^20^. *Cbr-daf-2* also has an RBFOX-binding motif near the 5′ splice site in the intron downstream of the cassette exon (Supplementary Fig. 1b). Exon 11.5 of *daf-2* resides in a similar position to a cassette exon, exon 11, of mammalian insulin receptor (IR) genes^20^. The RBFOX-binding motif also resides near the 5′ splice site in the intron downstream of exon 11 of the human and mouse IR genes. It was predicted that inclusion of *Insr* exon 11 is directly promoted by Rbfox proteins in the mouse brain^5^. The conserved regulation by the RBFOX family proteins in the nematodes and mammals suggests the biological importance of RBFOX-regulated alternative splicing of insulin receptors in the nervous systems.

Mammalian CELF family proteins have been shown to repress exon 11 of the IR genes^40^,^41^. These CELF proteins are widely expressed in numerous tissues, whereas alternative splicing of exon 11 of the IR genes occurs in a tissue-specific manner^42^. It was postulated that the coordination of CELF proteins and other factors, such as muscleblind-like 1, confer tissue-type specificity to the alternative splicing regulation of IR^43^. All six human CELF family genes are strongly expressed in the nervous system^6^. The CELF family proteins regulate cell-type-specific alternative splicing in concert with other splicing factors, including PTB^44^,^45^. These reports raise the possibility that the CELF family proteins and other splicing factors, such as RBFOX and PTB, might cooperatively control neuron-type-specific alternative splicing of the mammalian IR genes in a manner analogous to that in the *C. elegans* nervous system. Dysfunction of neuronal IR signalling is linked to neurological diseases such as Alzheimer's disease^46^. However, an isoform-specific function of IR in the brain is still unclear. It will be interesting to investigate alternative splicing patterns of the IR genes in distinct neuron types and an isoform-specific function of IR in the brain.

## Methods

### *C. elegans* strains

*C. elegans* Bristol strain N2 was used as the wild type. The strains were grown and maintained at 20 °C. Standard genetics methods were used to generate multiple mutants by crosses^47^. *hrp-1(ok963) IV* and *ptb-1(gk347274) II; asd-1(ok2299) III; fox-1(e2643) X* were maintained with the genetic balancers, *nT1[qIs51] (IV;V)* and *qC1[qIs26] III*, respectively. *daf-2(pe1230)* was previously isolated by forward genetic screening for mutants defective in taste-avoidance learning^20^. No *daf-2(pe1230)* worm shows a dauer constitutive (Daf-c) phenotype at 20 °C. The mutation site of *pe1230* is shown in Supplementary Table 1. The mutants and transgenic worms used in this study are listed in Supplementary Tables 2–4.

### DNA constructs and transgenesis

To generate *daf-2* cDNA expression plasmids, a PCR-amplified *H20* promoter (2.5 kb) was inserted upstream of *daf-2a/c* cDNAs in pBS-*daf-2a/c* plasmids^20^. Plasmids except for *daf-2* cDNA constructs were generated using the Gateway system (Invitrogen) as previously described^20^. To generate *daf-2* exon 11.5-splicing reporter constructs, a PCR-amplified genomic region of *daf-2* between parts of exons 11 and 12 was inserted upstream of *mRFP*, *EGFP* or *EGFP*-fused *mRFP* separated by a sequence with an acceptor site of a spliced leader (SL2), in a Gateway destination vector, pPD-DEST2. Mutations were introduced into exon 11.5, exon 12 and/or RBFOX-binding motifs by PCR reactions. Sequences of the *daf-2* genomic region and mutation sites in the splicing reporters are shown in Supplementary Fig. 1a. To generate Flag-tagged *pab-1* expression constructs, Flag-tagged *pab-1* cDNA was inserted upstream of an acceptor site of SL2 followed by *GFP* in pPD-DEST2. To generate *ptb-1* cDNA expression constructs, PCR-amplified *ptb-1a* and *ptb-1b* cDNAs were inserted upstream of an acceptor site of SL2 followed by *CFP* in pPD-DEST2. To generate a *spGFP1-10* expression construct, *spGFP1-10* cDNA was inserted into pPD-DEST2. To generate a *spGFP11::mCherry* expression construct, *spGFP11* cDNA was inserted in-frame upstream of *mCherry* cDNA in pPD-DEST2. *spGFP1-10* and *spGFP11* cDNAs are a generous gift from Dr Cori Bargmann. Gateway entry vectors with promoters for *H20* (ref. 21), *casy-1* (ref. 20), *gpc-1* (ref. 48), *glr-1* (ref. 19) and *odr-2* (ref. 19) were previously described. PCR-amplified *asd-1* (2.3 kb), *ptb-1a* (5.1 kb), *ptb-1b* (7.4 kb) and *eef-1A.1* (2.9 kb) promoters were cloned into a Gateway entry vector. P*fox-1::spGFP1-10* and P*fox-1::spGFP11::mCherry* were expressed as a vector-free construct generated by a PCR fusion-based method^49^. A 4.0-kb upstream regulatory sequence of *fox-1* was fused to *spGFP1-10* or *spGFP11::mCherry* followed by *unc-54* 3′-untranslated region using PCR fusion reactions. Germline transformations were performed using standard microinjection methods^50^. DNA constructs were injected at concentrations between 20 and 100 ng μl^−1^ and with or without a co-injection marker, *unc-122p::mCherry* (20 ng μl^−1^), pRF4 carrying *rol-6(su1006)* (50 ng μl^−1^) or *myo-3p::Venus* (10 or 30 ng μl^−1^), and a carrier DNA, pPD49.26. Injection mixtures were prepared at a final concentration of 100 ng μl^−1^.

### Cloning of *ptb-1* and *Cbr-daf-2* cDNAs

Full-length *ptb-1* and partial *Cbr-daf-2* cDNAs were amplified by RT–PCR from *C. elegans* and *C. briggsae* total RNAs, respectively. The *ptb-1* and *Cbr-daf-2* cDNAs were cloned and sequenced to examine the genomic structures. The *ptb-1a* and *ptb-1b* cDNA sequences were submitted to DNA Data Bank of Japan and were given accession numbers, LC026472 and LC026473, respectively. Primers used for PCR reactions are listed in Supplementary Table 5.

### mRNA tagging

Neuron-type-specific poly(A)^+^ RNA was isolated from transgenic adult worms with FLAG-PAB-1 expressed under a neuron-type-selective promoter according to our previously published protocol^51^ with some modifications. We used transgenic worms expressing FLAG-PAB-1 and GFP under the *gpc-1* or *glr-1* promoter (Fig. 2a–c; Supplementary Tables 3 and 4). To crosslink poly(A)^+^RNA with FLAG-PAB-1, the worms grown on nematode growth medium (NGM) plates were collected and washed three times with M9 buffer, and then treated with 5% formaldehyde in M9 for 20 min at room temperature. To immunoprecipitate the RNA/FLAG-PAB-1 complexes, the supernatant of worm lysates was incubated with anti-FLAG M2 affinity gel beads (Sigma-Aldrich) for 4 h at 4 °C. The precipitated materials including RNA/FLAG-PAB-1 complexes were incubated for 30 min at 65 °C to remove the crosslink.

### RT–PCR and quantitative RT–PCR assays

Neuron-type-specific RNA and total RNA were reverse-transcribed, and then subjected to PCR reactions with gene- or exon-specific primers to compare transcript levels between samples. The RT–PCR products were analysed using the microchip electrophoresis system, MultiNA (Shimadzu) or BioAnalyzer (Agilent), and each analysis was performed in quadruplicate (Fig. 2f) or triplicate (Fig. 6a). Images have been cropped for presentation. Full-size images are presented in Supplementary Figs 10–12. Real-time PCR analyses were performed using a Thermal Cycler Dice Real Time System (Takara). Serial dilutions of *daf-2a/c* cDNA or cDNA prepared from total RNA of wild-type worms were used to generate a standard curve. A ubiquitous gene, *eef-1A.1*, was used as an internal standard and each analysis was performed in triplicates. Primers are listed in Supplementary Table 5.

### Quantitative analysis of confocal images

Anaesthetized worms at the L1/L2 stage were imaged on a 5% agarose pad. Note that there was no significant difference in expression patterns of the neuronal *daf-2* exon 11.5-skipping/inclusion reporters between wild-type L1 and L2 worms (Supplementary Fig. 13). Images of the head regions of ⩾20 individual worms carrying *daf-2* exon 11.5-splicing reporters were acquired for each genotype. Z-series images (slice spacing of 1 μm) were acquired with a Leica SP5 confocal microscope using a × 63/1.30 objective. The same parameter settings were used to acquire all images for quantification. To determine fluorescence intensity ratios, z-stack images were created using a sum intensity projection in ImageJ. After background subtraction, averaged fluorescence intensities in a region of interest around the head ganglia were obtained in the z-stack images, and RFP-to-GFP intensity ratios were determined (Fig. 3a). The intensity ratios were normalized by the averaged values obtained for wild-type or control worms, which were acquired in a set of experiments within 1 day. To determine the fractions of GFP and/or RFP expression in the head ganglia, the z-stack images were cropped to include only the head ganglia and their binary images were created using ImageJ. The IsoData algorithm was applied to all images of wild-type worms acquired in a set of experiments to determine a threshold value, and the average of these values was used to binarise all images acquired in the set of experiments. Pixel fractions with GFP alone, RFP alone or both signals were calculated in the z-series binary images using an R script that counts the total numbers of each type of pixels (that is, pixels with GFP alone, RFP alone or both signals) within all pixels of the z-series binary images and calculates the fractions of each pixel type (Fig. 3a).

### Salt concentration learning assay

Salt concentration learning assays were performed according to our previously reported procedure^33^ with some modifications. For conditioning, adult worms were transferred to NGM plates with 25 or 100 mM NaCl for 5 h. After conditioning, the worms were placed at the centre of a test plate with a NaCl gradient and were allowed to crawl for 45 min. Chemotaxis and learning indices were determined as shown in Fig. 10a. A learning index after conditioning under fed conditions was determined by subtracting the chemotaxis index after conditioning on an NGM plate with 25 mM NaCl from that with 100 mM NaCl. A learning index after conditioning under starvation conditions was determined by subtracting the chemotaxis index after conditioning on an NGM plate with 100 mM NaCl from that with 25 mM NaCl.

## Additional information

**How to cite this article:** Tomioka, M. *et al*. Splicing factors control *C. elegans* behavioural learning in a single neuron by producing DAF-2c receptor. *Nat. Commun.* 7:11645 doi: 10.1038/ncomms11645 (2016).

## Supplementary Material

Supplementary Information Supplementary Figures 1-13 and Supplementary Tables 1-5 and Supplementary References.

Supplementary Movie 1 Z-series of confocal images of a larva expressing the exon 11.5-skipping/inclusion reporters under the H20 promoter.
