# Supplementary material for: Splicing factors control C. elegans behavioural learning in a single neuron by producing DAF-2c receptor
Source: Nat Commun. 2016 May 20;7:11645. doi: 10.1038/ncomms11645 (PMC4876481; doi:10.1038/ncomms11645)
Supplement: Supplementary Information — Supplementary Figures 1-13 and Supplementary Tables 1-5 and Supplementary References. [file ncomms11645-s1.pdf]

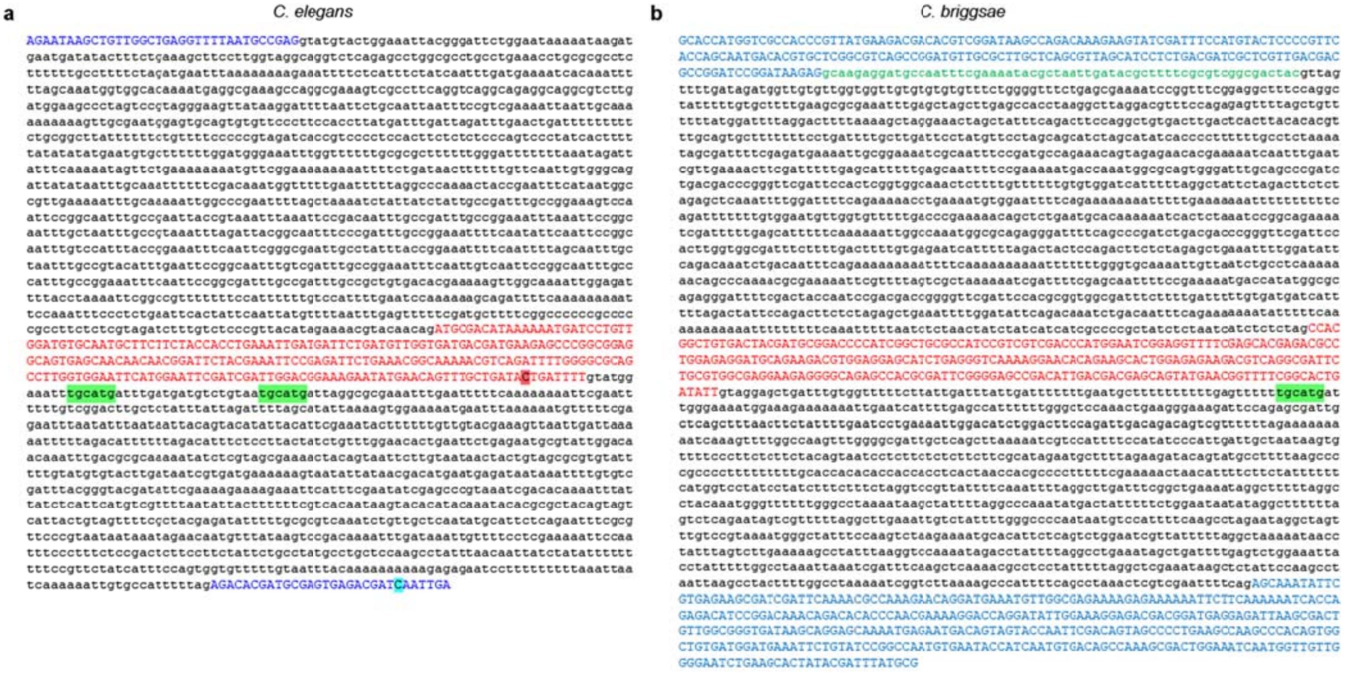

Supplementary Figure 1. Cassette exons and their flanking sequences of *daf-2* and *Cbr-daf-2*. (**a**) A *daf-2* genomic sequence used for exon 11.5-splicing reporters. Parts of exons 11 and 12 are in blue, and exon 11.5 is in red. One-base pair insertion and deletion in exon 11.5 and exon 12 are shaded in red and blue, respectively. (**b**) A genomic sequence spanning from exon 16 to exon 17 of the *Cbr-daf-2* gene. Constitutive exons, exons 16 and 17, and a cassette exon (named as exon 16.5) are in blue and red, respectively. Extension of exon 16 is in green. The RBFOX-binding motif is shaded in green.

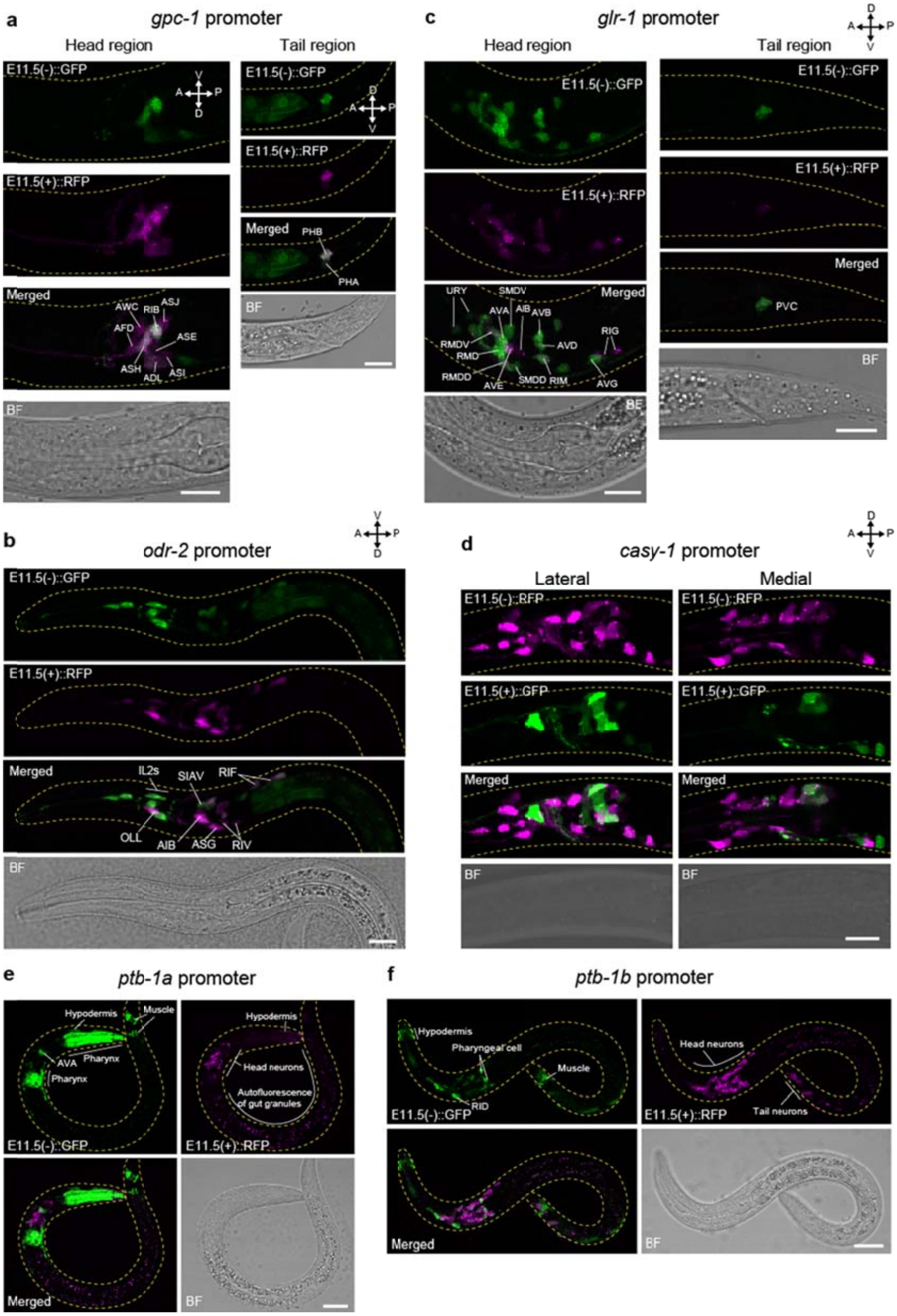

a
gpc-1 promoter
Head region
Tail region
E11.5(-)::GFP
E11.5(+)::RFP
Merged
AWC
RIB
ASJ
AFD
ASE
ASH
ADL
ASI
PHB
PHA
BF
b
odr-2 promoter
IL2s
SIAV
RIF
OLL
AIB
ASG
RIV
c
glr-1 promoter
URY
SMDV
AVA
AIB
AVB
RMDV
RMD
AVD
RIG
RMDD
AVE
SMDD
RIM
AVG
PVC
d
casy-1 promoter
Lateral
Medial
E11.5(-)::RFP
E11.5(+)::GFP
e
ptb-1a promoter
Hypodermis
Muscle
AVA
Pharynx
Head neurons
Autofluorescence of gut granules
f
ptb-1b promoter
Pharyngeal cell
RID
Tail neurons
A
P
D
V

Supplementary Figure 2. **Neuron-class-specific alternative splicing of *daf-2* exon 11.5.** (**a-c, e** and **f**) Expression patterns of the alternative splicing reporters of *daf-2* exon 11.5. The reporter minigenes shown in Figure 1a were expressed under the *gpc-1* promoter (**a**), the *odr-2* promoter (**b**), the *glr-1* promoter (**c**), the *ptb-1a* promoter (**e**) and the *ptb-1b* promoter (**f**). Predominant RFP signals are observed in the amphid sensory neurons (AWC, AFD, ASJ, ASH, ASE, ADL, ASI and ASG), OLL sensory neurons, AIB interneurons and RIV head motor neurons (**a** and **b**) predominant GFP signals are observed in command interneurons (AVA, AVB, AVD, and PVC), AVG interneurons and SMD and RMD head motor neurons (**c**). (**d**) Expression patterns of alternative splicing reporters of *daf-2* exon 11.5, in which the fluorescent protein cDNAs are swapped between the reporter minigenes shown in Figure 1a. The reporters were expressed under the *casy-1* promoter. L1/L2 larvae are shown. Scale bars, 10 μm (**a-d**); 20 μm (**e**, **f**).

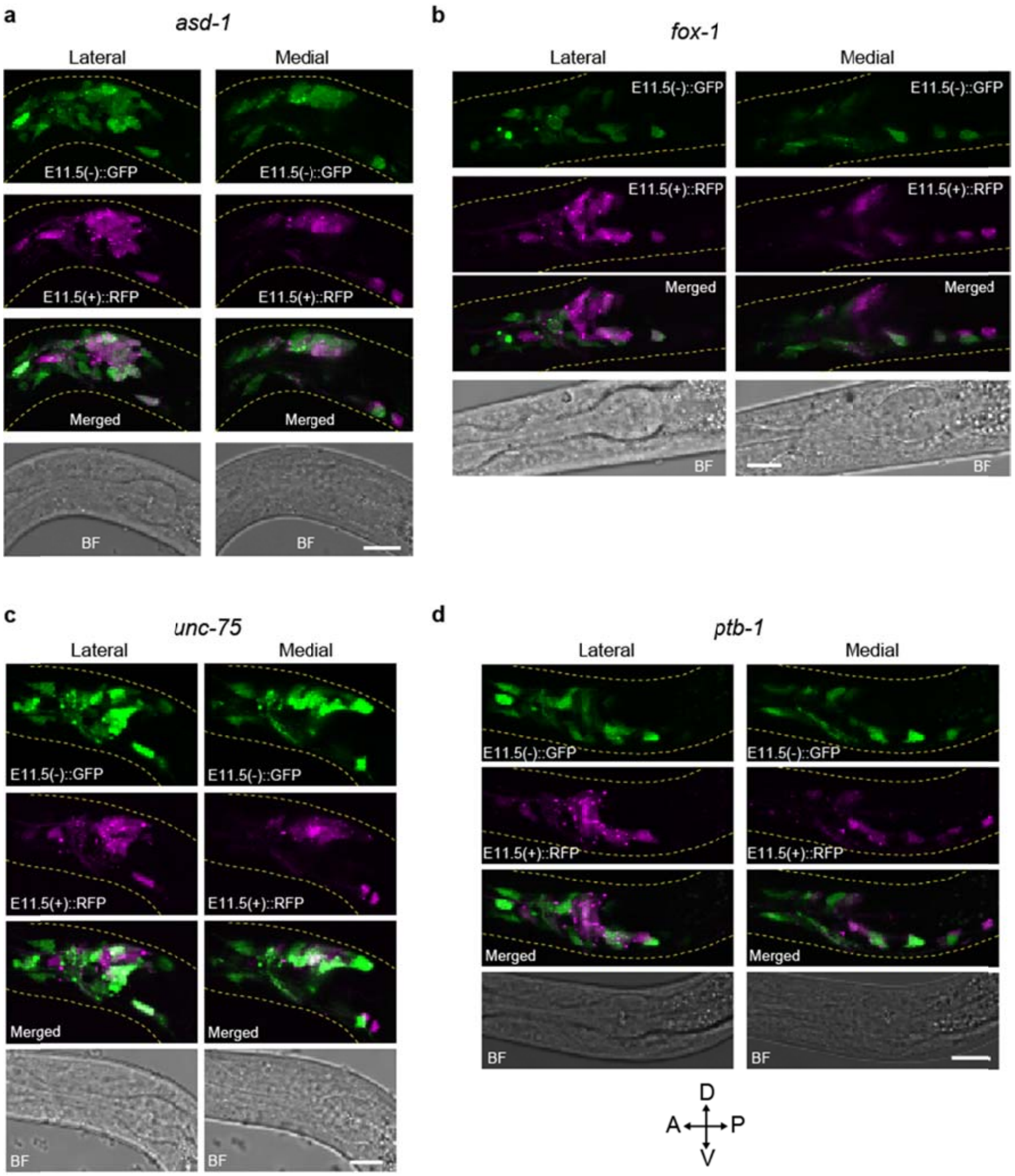

Supplementary Figure 3. **Expression patterns of the neuronal exon 11.5-skipping/inclusion reporter in *unc-75, ptb-1* and the RBFOX single mutants**. Maximum intensity projection images of the neuronal exon 11.5-skipping/inclusion reporter in the *asd-1(ok2299)* (**a**), *fox-1(e2643)* (**b**), *unc-75(e950)* (**c**) and *ptb-1(gk347274)* (**d**) mutants. The head regions of L1/L2 larvae are shown. Scale bars, 10 μm.

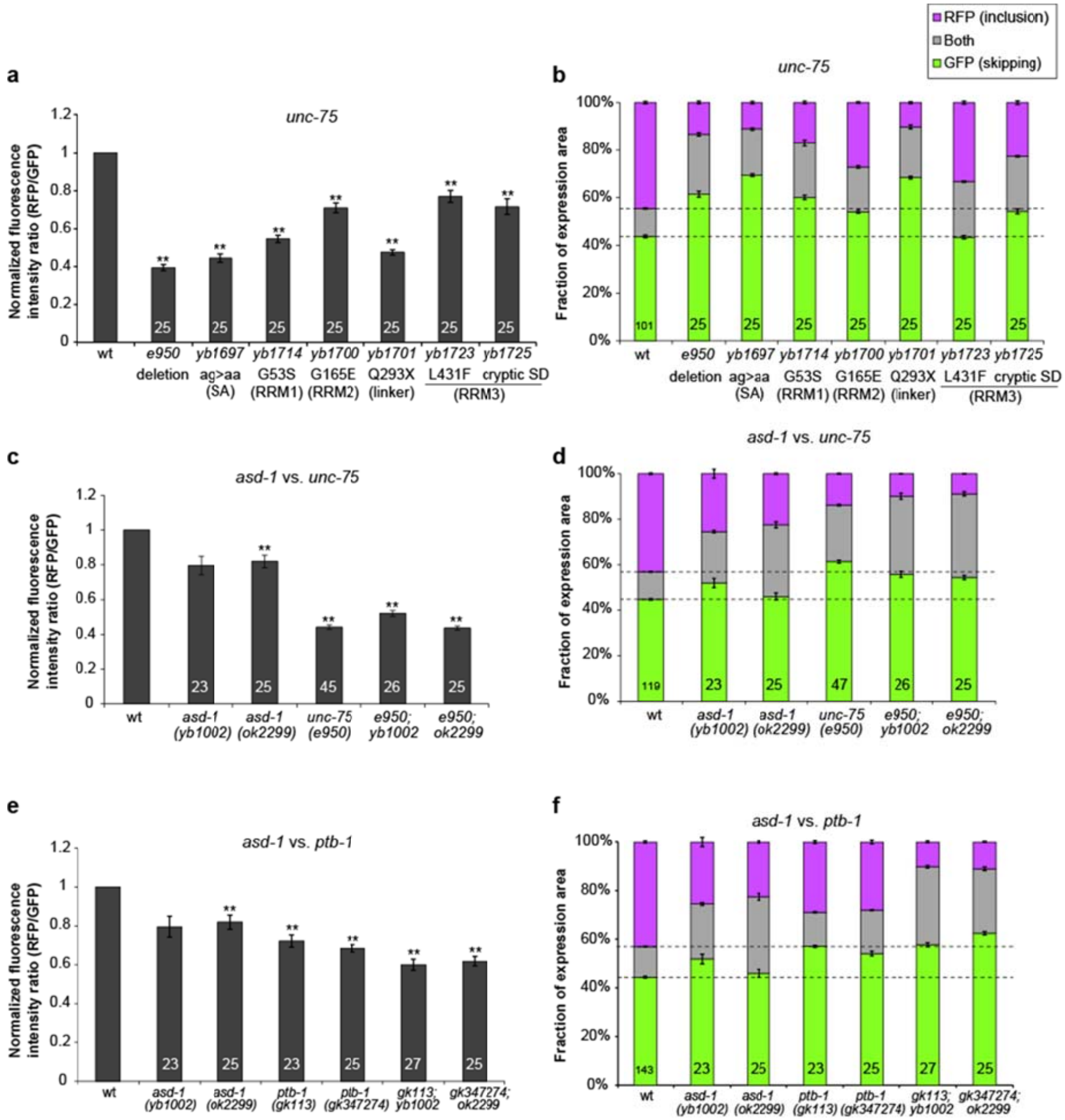

Supplementary Figure 4. **Effects of *unc-75* mutations and genetic interaction analyses in *daf-2* exon 11.5 selection.** (**a** and **b**) Alternative splicing analyses of *daf-2* exon 11.5 in *unc-75* mutants. The positions and consequences of the mutations are shown below the allele names. SA, splice acceptor; SD, splice donor. (**c**-**f**) Genetic interactions of *asd-1* with *unc-75* (**c** and **d**) and with *ptb-1* (**e** and **f**) in alternative splicing of *daf-2* exon 11.5. Normalised fluorescence intensity ratios of RFP to GFP (**a**, **c**, **e**) and fractions of GFP and RFP expression (**b**, **d**, **f**) of the neuronal exon 11.5-skipping/inclusion reporter in the wild-type and mutant worms. Data were normalised by the averaged values in the wild-type worms (n =25, **a**; $n \geq 22$, **c** and **e**). The *n* values are shown in each bar. Error bars represent s.e.m. $^{**}p<0.01$, different from wild type, two-tailed *t*-test with Bonferroni correction.

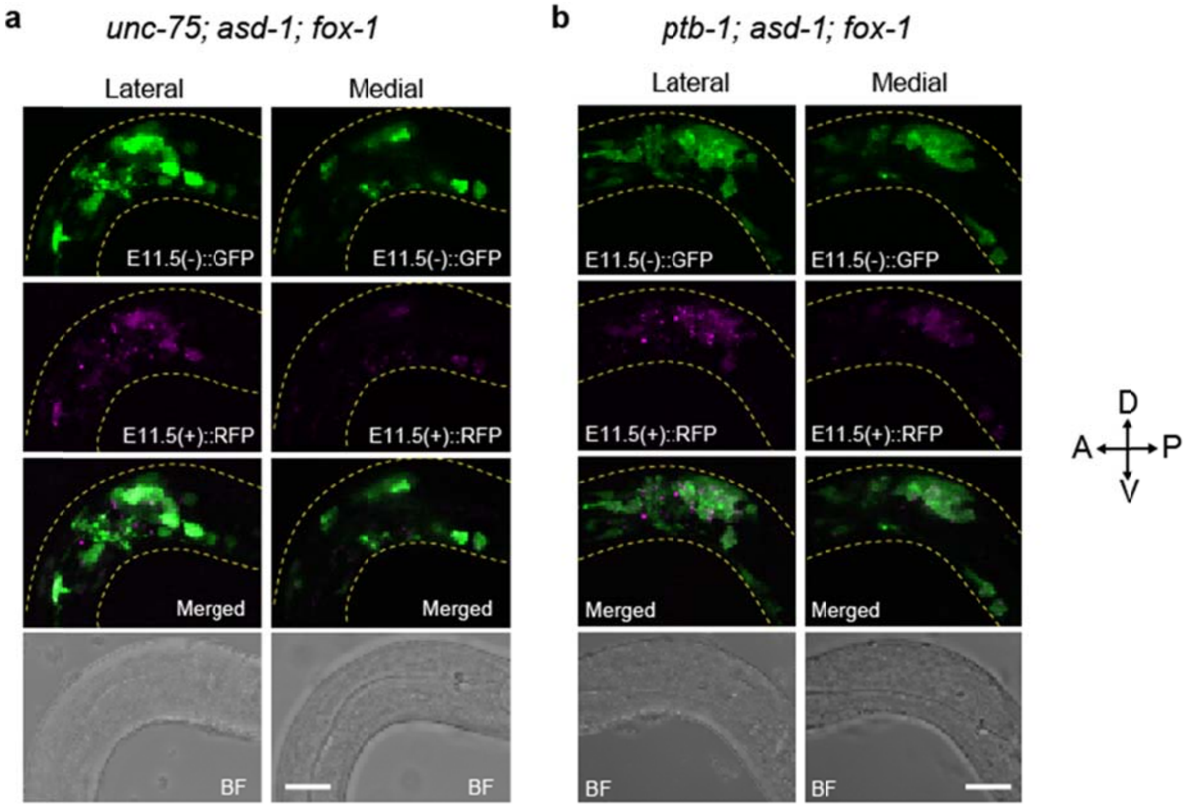

Supplementary Figure 5. **Expression patterns of the neuronal exon 11.5-skipping/inclusion reporter in triple mutants**. Maximum intensity projection images of the neuronal exon 11.5-skipping/inclusion reporter in the *unc-75(e950); asd-1(ok2299); fox-1(e2643)* (**a**) and *ptb-1(gk347274); asd-1(ok2299); fox-1(e2643)* (**b**) mutants. The head regions of L1/L2 larvae are shown. Scale bars, 10 µm.

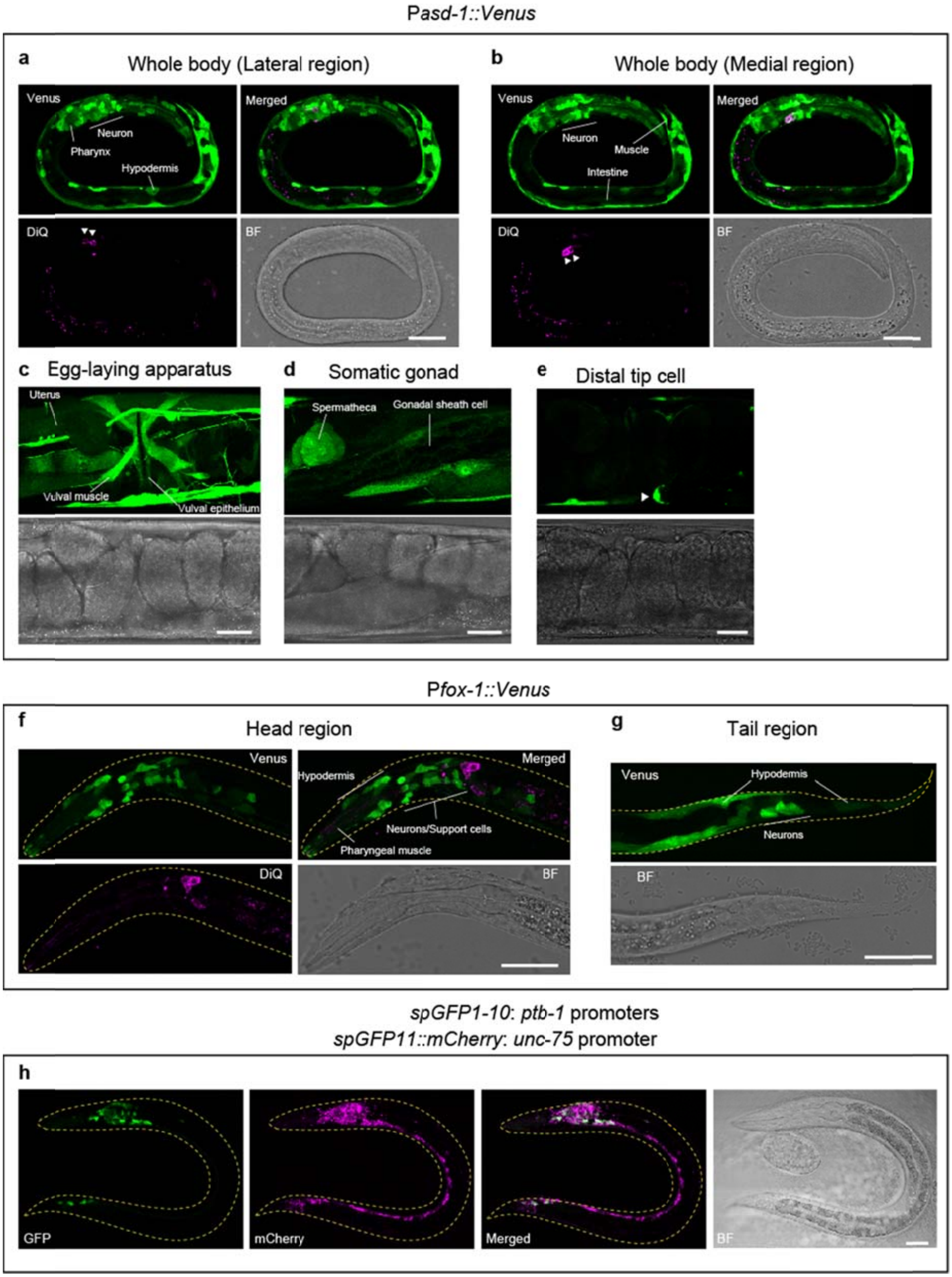
Pasd-1::Venus
a
Whole body (Lateral region)
Venus
Neuron
Pharynx
Hypodermis
Merged
DiQ
BF
b
Whole body (Medial region)
Venus
Neuron
Muscle
Intestine
Merged
DiQ
BF
c
Egg-laying apparatus
Uterus
Vulval muscle
Vulval epithelium
d
Somatic gonad
Spermatheca
Gonadal sheath cell
e
Distal tip cell
Pfox-1::Venus
f
Head region
Venus
Merged
Hypodermis
Neurons/Support cells
Pharyngeal muscle
DiQ
BF
g
Tail region
Venus
Hypodermis
Neurons
BF
spGFP1-10: ptb-1 promoters
spGFP11::mCherry: unc-75 promoter
h
GFP
mCherry
Merged
BF

Supplementary Figure 6. **Expression patterns of the RBFOX family genes and co-expression analysis with *unc-75* and *ptb-1* genes.** (**a**-**e**) Expression patterns of *Venus* driven by the regulatory sequence including 2-kb of upstream sequence through exon 2 of the *asd-1* gene. The whole body of an L1 larva (**a** and **b**) and mid-body regions of adults (**c**-**e**) are shown. (**f** and **g**) Expression patterns of *Venus* driven by the regulatory sequence including 5-kb of upstream sequences through exon 3 of the *fox-1* gene. The head (**f**) and tail (**g**) regions of an L1 larva are shown. Some amphid sensory neurons are stained with a lipophilic dye, DiQ (**a**, **b** and **f**). (**h**) Maximum intensity projection images of worms expressing *spGFP1-10* and *spGFP11::mCherry* by the *ptb-1* (*ptb-1a* and *ptb-1b*) promoters and the *unc-75* promoter, respectively. The whole body of an L2 larva is shown. Scale bars, 20 µm.

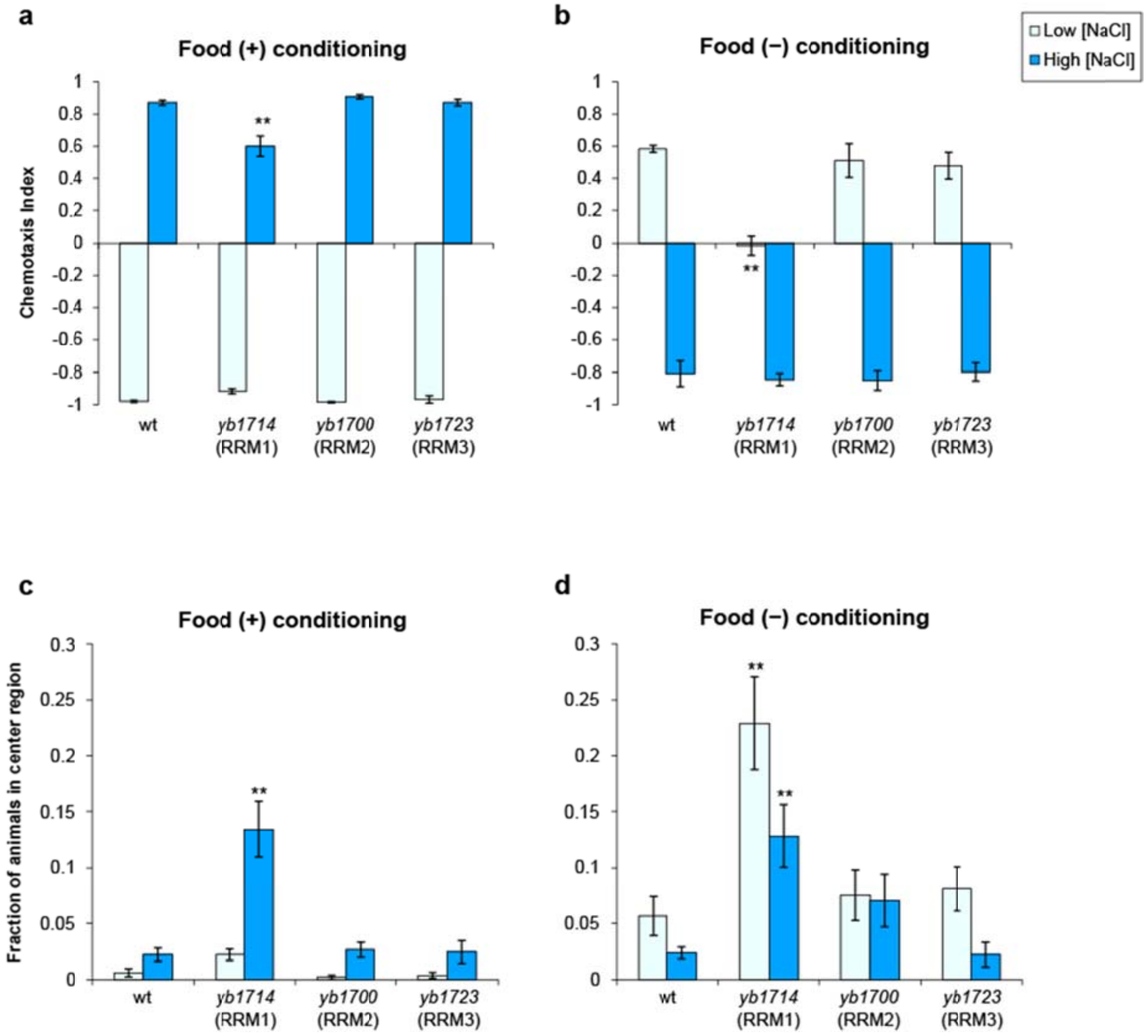

Supplementary Figure 7. **Salt concentration learning of reduction-of-function alleles of the *unc-75* mutants.** (**a**-**d**) Chemotaxis indices (**a** and **b**) and fractions of worms in the centre region (region C in Figure 10a), where worms with reduced motility remain, (**c** and **d**) are shown. Salt conditioning was performed under fed (**a** and **c**) or starvation (**b** and **d**) conditions. n ≥ 4 assays. Error bars represent s.e.m. **p<0.01, different from wild-type worms, one-way ANOVA followed by Dunnett post hoc test.

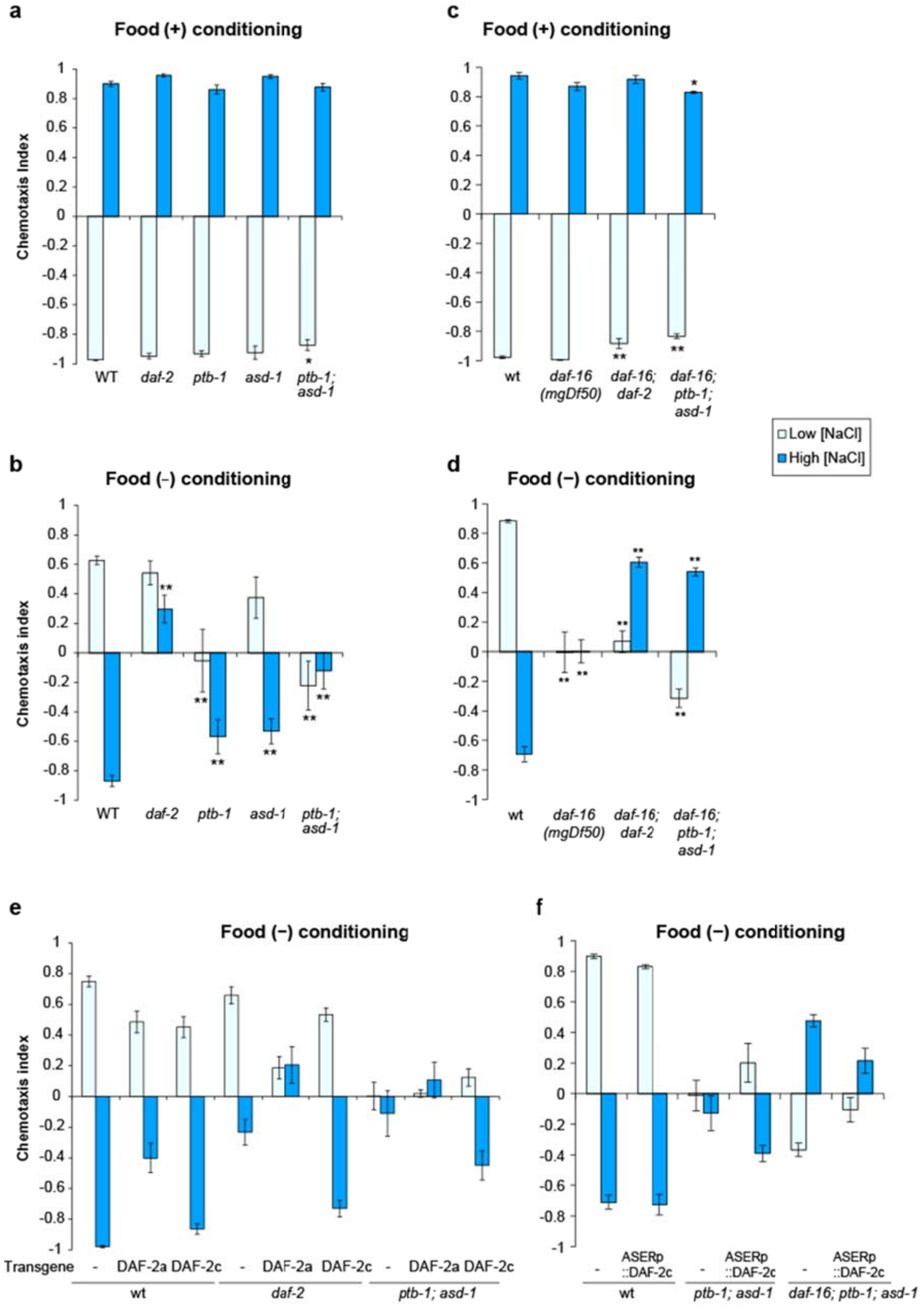
a
Food (+) conditioning
Chemotaxis Index
WT
daf-2
ptb-1
asd-1
ptb-1; asd-1
b
Food (-) conditioning
Chemotaxis index
WT
daf-2
ptb-1
asd-1
ptb-1; asd-1
c
Food (+) conditioning
wt
daf-16 (mgDf50)
daf-16; daf-2
daf-16; ptb-1; asd-1
Low [NaCl]
High [NaCl]
d
Food (−) conditioning
wt
daf-16 (mgDf50)
daf-16; daf-2
daf-16; ptb-1; asd-1
e
Food (−) conditioning
Chemotaxis index
Transgene
-
DAF-2a
DAF-2c
wt
daf-2
ptb-1; asd-1
f
Food (−) conditioning
ASERp ::DAF-2c
wt
ptb-1; asd-1
daf-16; ptb-1; asd-1

Supplementary Figure 8. **DAF-2c expression in ASER relieves impaired taste-avoidance learning of the RBP mutant.** Chemotaxis indices of worms after salt conditioning are shown. (**a** and **b**) Chemotaxis indices of the wild type, *daf-2(pe1230), ptb-1(gk347274), asd-1(ok2299)* and *ptb-1(gk347274); asd-1(ok2299)* mutants after salt conditioning under fed (**a**) or starvation (**b**) conditions. (**c** and **d**) Chemotaxis indices of the wild type, *daf-16(mgDf50)*, *daf-16(mgDf50); daf-2(e1370)* and *daf-16(mgDf50); ptb-1(gk347274); asd-1(ok2299)* after salt conditioning under fed (**c**) or starvation (**d**) conditions. (**e**) Chemotaxis indices of wild-type, *daf-2(pe1230)* and *ptb-1(gk347274); asd-1(ok2299)* worms expressing none (−) or either of the DAF-2 isoforms under the *H20* promoter. Salt conditioning was performed under starvation conditions. (**f**) Chemotaxis indices of wild-type, *ptb-1(gk347274); asd-1(ok2299)* and *daf-16(mgDf50); ptb-1(gk347274); asd-1(ok2299)* worms expressing none (−) or DAF-2c in ASER under the *gcy-5* promoter. Salt conditioning was performed under starvation conditions. n ≥ 4 assays (**a**-**d**). n ≥ 6 assays (**e** and **f**). Error bars represent s.e.m. *$p<0.05$; **$p<0.01$, different from wild-type or control worms, one-way ANOVA followed by Dunnett post hoc test.

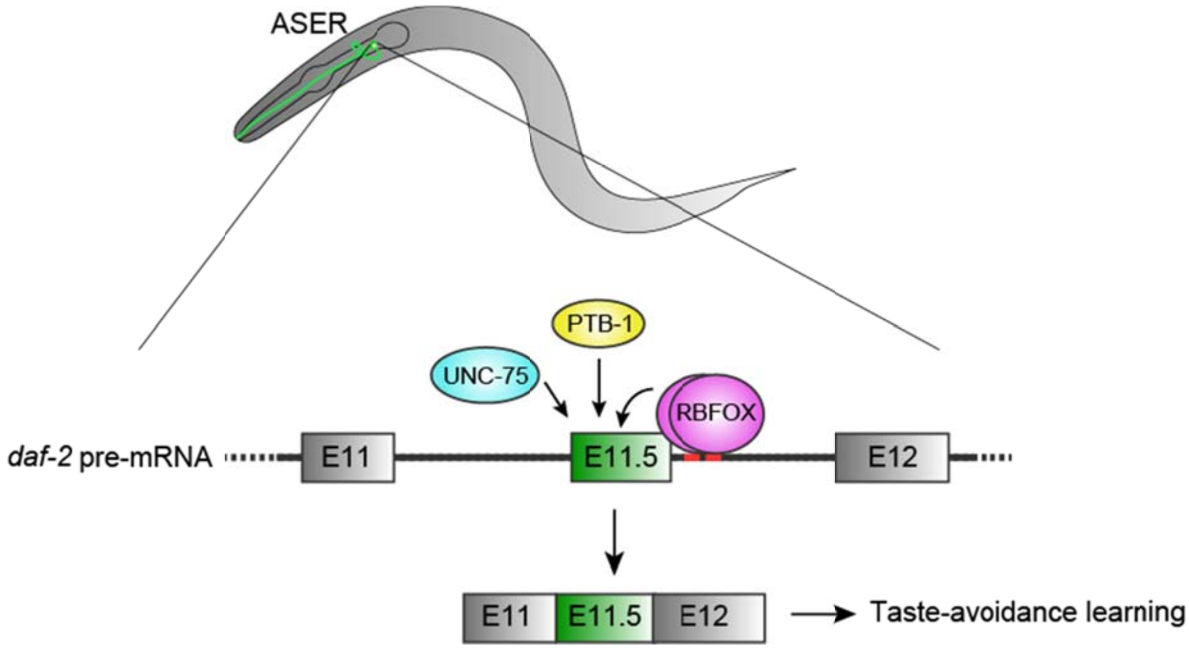

Supplementary Figure 9. **Neuron-class-specific inclusion of *daf-2* exon 11.5 finely regulated by evolutionarily conserved RBPs underlies taste-avoidance learning.** RBFOX family proteins, ASD-1 and FOX-1, directly activate exon 11.5 inclusion. Cooperative action of PTB-1 (polypyrimidine tract-binding protein orthologue) and UNC-75 (CELF family protein) with the RBFOX family proteins is required for preferential inclusion of *daf-2* exon 11.5 in the taste receptor neuron ASER in taste-avoidance learning.

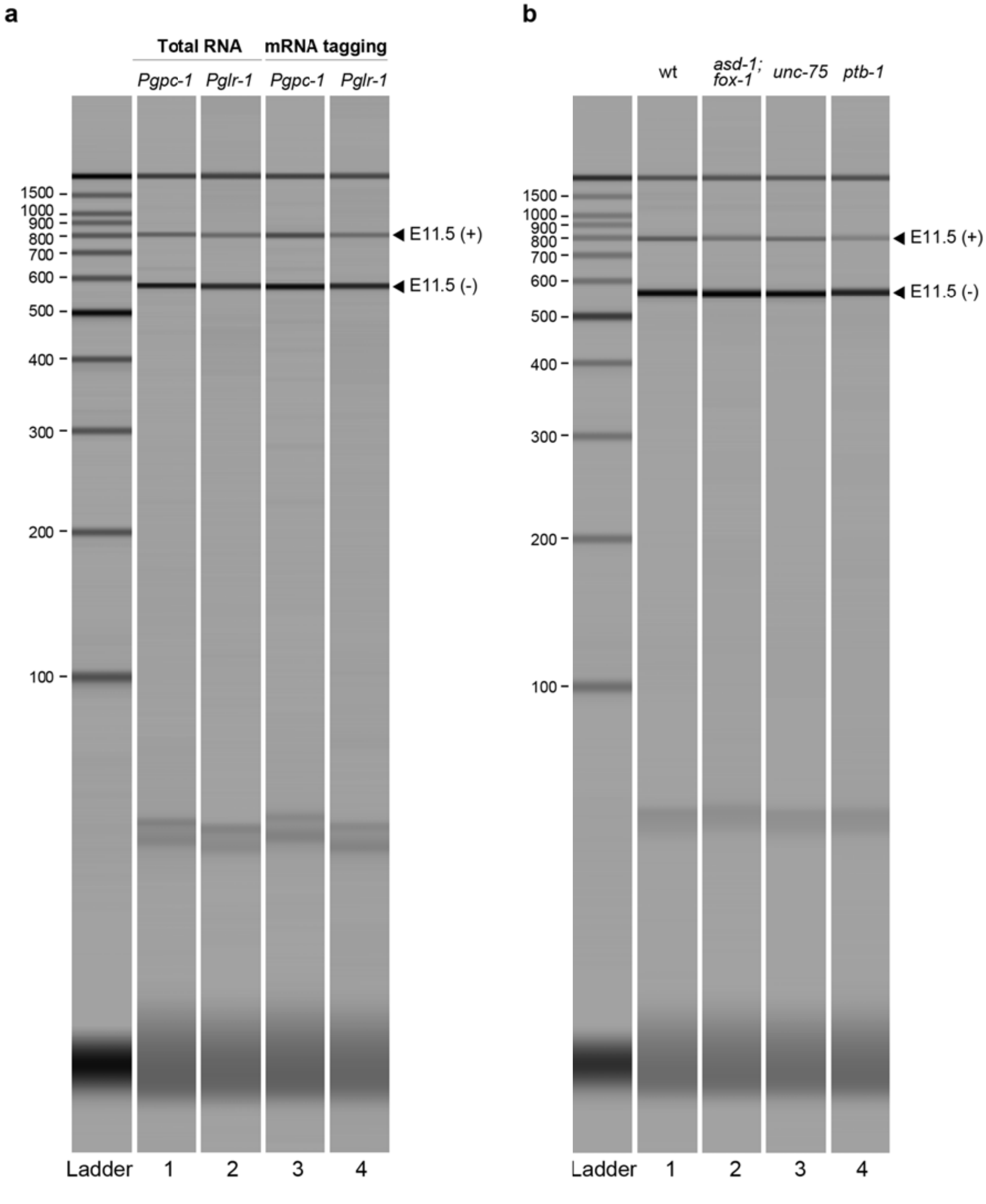

Supplementary Figure 10. **RT-PCR analysis of *daf-2* exon 11.5.** Full-size electrophoresis images of RT-PCR samples shown in Fig. 2f (**a**) and 6a (**b**). The RT-PCR products were analysed using the microchip electrophoresis system, MultiNA (Shimadzu).

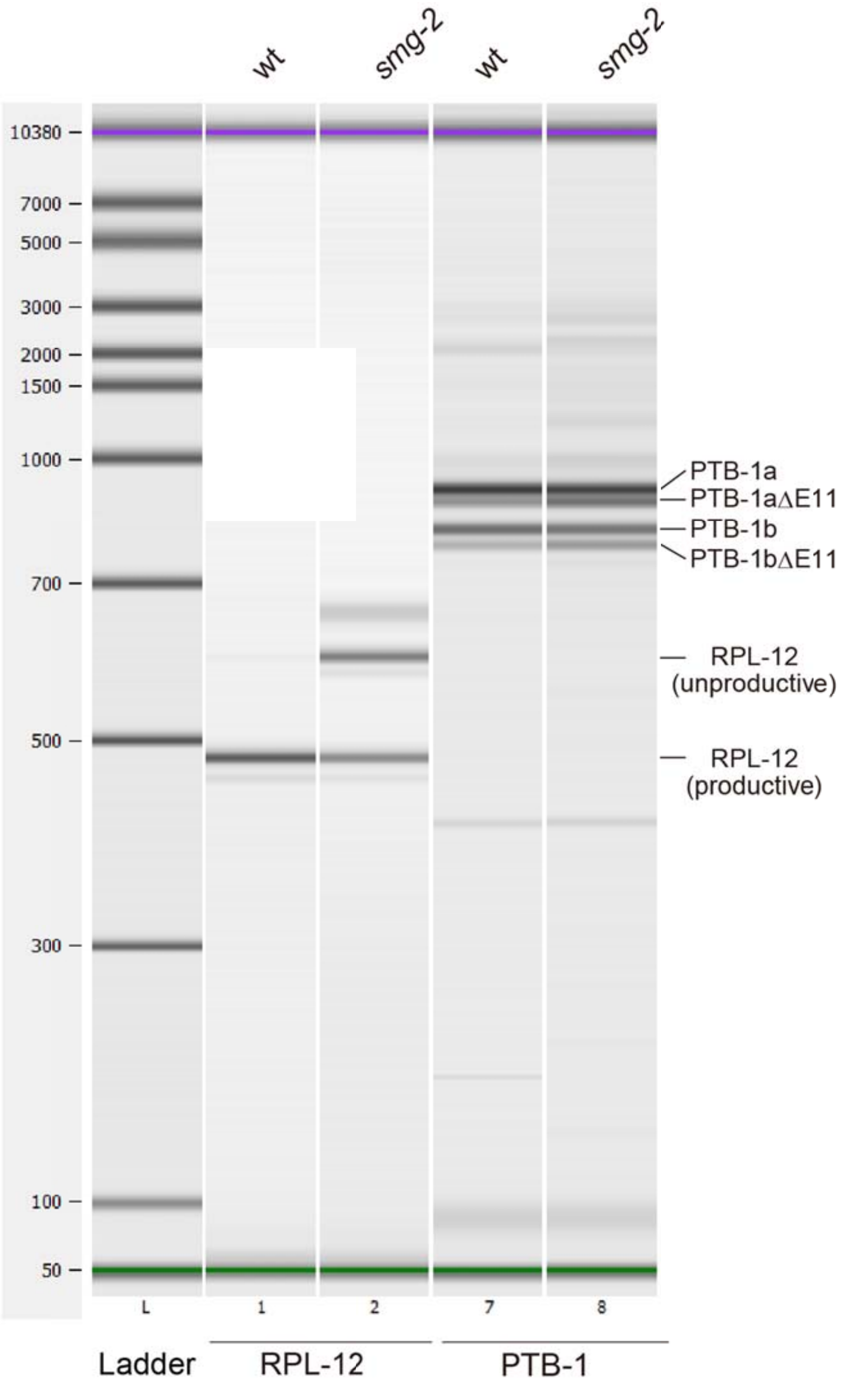

Supplementary Figure 11. **Full-size electrophoresis images of RT-PCR samples shown in Fig. 8b.** RT-PCR analyses of PTB-1 and RPL-12 transcripts. The RT-PCR products were analysed using the microchip electrophoresis system, BioAnalyzer (Agilent).

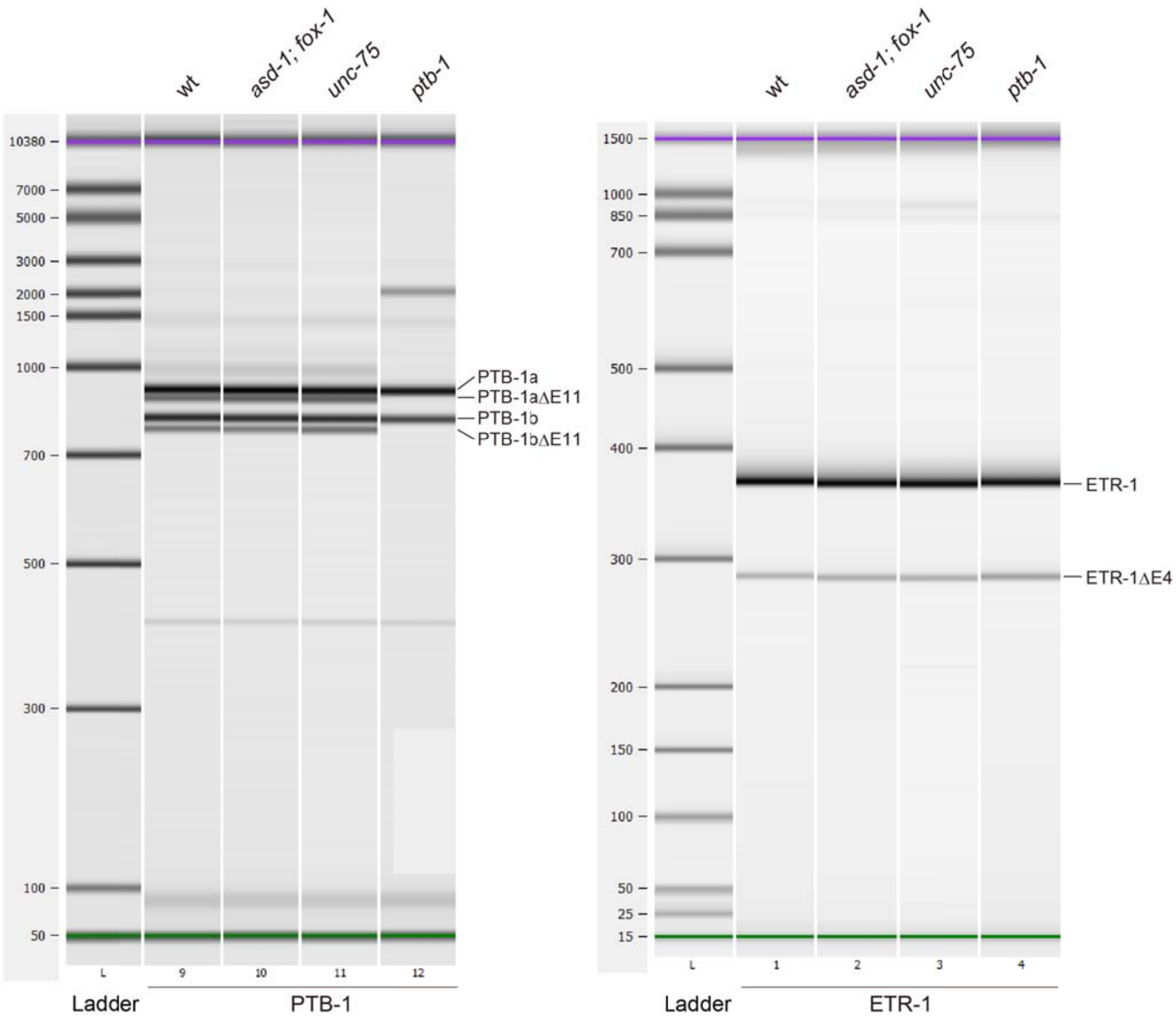

Supplementary Figure 12. **Full-size electrophoresis images of RT-PCR samples shown in Fig. 8c.** RT-PCR analyses of PTB-1 and ETR-1 transcripts. The RT-PCR products were analysed using the microchip electrophoresis system, BioAnalyzer (Agilent).

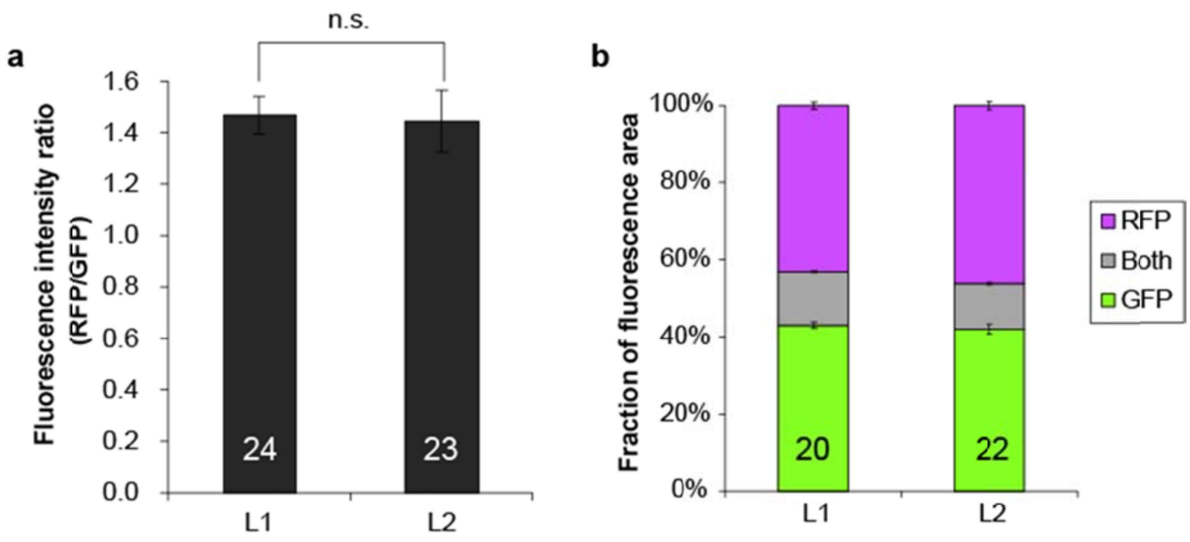

Supplementary Figure 13. **Expression patterns of the neuronal exon 11.5-skipping/inclusion reporter did not significantly change between L1 and L2 stages.** Fluorescence intensity ratios of RFP to GFP (**a**) and fractions of GFP and RFP expression (**b**) of the neuronal exon 11.5-skipping/inclusion reporter at the L1 and L2 stages in the wild-type background. The *n* values are shown in each bar. Error bars represent s.e.m. n.s., not significant (two-tailed *t*-test).

Supplementary Table 1. List of mutant alleles

LG	Gene	Human homolog	Allele	Mutation	Ref.
I	*unc-75*	CELF /BRUNOL	*e950*	Deletion of upstream and a part of *unc-75* (exons 1-5)	1
			yb1714	Missense mutation in RRM1, G53S	2
			yb1697	Splicing acceptor site of intron 2	
			yb1700	Missense mutation in RRM2, G165E	
			yb1701	Nonsense mutation in divergent domain, Q293X	
			yb1723	Missense mutation in RRM3, L431F	
			yb1725	Splicing donor site of intron 8	
	hrpf-1	hnRNP F/H	*tm3406*	426bp deletion (removing a part of exons 3-4)	3
II	*sym-2*		*gk150369*	Nonsense mutation R11X	4
	ptb-1	PTB	*gk113*	542bp deletion (removing a part of RRM3 and RRM4)	3
			gk347274	Nonsense mutation Q347X	4
	exc-7	HuC/D	*ok370*	1404bp deletion	3
III	*rsp-8*	TRA2β	*tm5638*	294bp deletion	
	asd-1	RBFOX	*yb1002*	Nonsense mutation Q53X	5
			ok2299	1611bp deletion (removing RRM)	
			yb978	Missense mutation in RRM, G140R	5
	daf-2	Insulin/IGF receptor	*e1370*	Missense mutation, P1465S	6
			pe1230	Splicing donor site of intron 5	7
IV	*hrp-1*	hnRNP A1	*ok963*	843bp deletion	3
V	*Y59A8B.10*	NOVA	*gk445934*	Splicing acceptor site of intron 2	4
X	*fox-1*	RBFOX	*e2643*	1255bp deletion	8

Supplementary Table 2. List of mutant strains

Strain	Genotype
CB950	*unc-75(e950) I*
KH1768	*unc-75(yb1700) I*
KH1752	*unc-75(yb1701) I*
KH1763	*unc-75(yb1714) I*
JN1755	*unc-75(yb1723) I*
GR1307	*daf-16(mgDf50) I*
VC20581	*ptb-1(gk347274) II*
VC176	*exc-7(ok370) II*
VC2118	*asd-1(ok2299) III*
KH1752	*asd-1(yb978) III*
JN1230	*daf-2(pe1230) III*
KH1234	*asd-1(yb978) III; fox-1(e2643) X*
JN1756	*ptb-1(gk347274) II; asd-1(ok2299) III*
JN735	*daf-16(mgDf50) I; daf-2(e1370) III*
JN1760	*daf-16(mgDf50) I; ptb-1(gk347274) II; asd-1(ok2299) III*

Supplementary Table 3. List of transgenic strains (wild-type background)

Strain	Genotype	Used for
JN1737	*peEx1737[eef-1A.1p::daf-2 E11-E11.5(+c)-E12::EGFP, eef-1A.1p::daf-2 E11-E11.5(+c)-E12(-c)::mRFP, rol-6(d)]*	Analyses of exon 11.5 skipping/inclusion of *daf-2*
JN1736	*peEx1736[H20p::daf-2 E11-E11.5(+c)-E12::EGFP, H20p::daf-2 E11-E11.5(+c)-E12(-c)::mRFP, rol-6(d)]*	
JN785	*peIs785[casy-1p::daf-2 E11-E11.5(+c)-E12::EGFP, casy-1p::daf-2 E11-E11.5(+c)-E12(-c)::mRFP]*	
JN1705	*peIs1705[gpc-1p::daf-2 E11-E11.5(+c)-E12::EGFP, gpc-1p::daf-2 E11-E11.5(+c)-E12(-c)::mRFP, rol-6(d)]*	
JN1738	*peEx1738[glr-1p::daf-2 E11-E11.5(+c)-E12::EGFP, glr-1p::daf-2 E11-E11.5(+c)-E12(-c)::mRFP, rol-6(d)]*	
JN1739	*peEx1739[odr-2p::daf-2 E11-E11.5(+c)-E12::EGFP, odr-2p::daf-2 E11-E11.5(+c)-E12(-c)::mRFP, rol-6(d)]*	
JN786	*peEx786[casy-1p::daf-2 E11-E11.5(+c)-E12(-c)::EGFP, casy-1p::daf-2 E11-E11.5(+c)-E12::mRFP]*	
JN1744	*peEx1744[ptb-1ap::daf-2 E11-E11.5(+c)-E12::EGFP, ptb-1ap::daf-2 E11-E11.5(+c)-E12(-c)::mRFP]*	
JN1745	*peEx1745[ptb-1bp::daf-2 E11-E11.5(+c)-E12::EGFP, ptb-1bp::daf-2 E11-E11.5(+c)-E12(-c)::mRFP]*	
JN1709	*peIs1709[gpc-1p::flag::pab-1::sl2::nls:GFP, unc-122p::mCherry]*	mRNA tagging
JN1710	*peIs1710[glr-1p::flag::pab-1::sl2::nls:GFP, unc-122p::mCherry]*	
JN1707	*peEx1707[gpc-1p::daf-2 E11-E11.5(+c)-E12(-c)::mRFP::SL2::EGFP]*	Analyses of exon 11.5 inclusion of *daf-2*
JN1708	*peEx1708[gpc-1p::daf-2 E11-E11.5(+c)-E12(-c)::mRFP::SL2::EGFP (M1/2)]*	
JN1727	*peEx1727[gpc-1p::daf-2 E11-E11.5(+c)-E12(-c)::mRFP::SL2::EGFP (M1/2)]*	
JN1728	*peEx1728[gpc-1p::daf-2 E11-E11.5(+c)-E12(-c)::mRFP::SL2::EGFP (M1)]*	
JN1729	*peEx1729[gpc-1p::daf-2 E11-E11.5(+c)-E12(-c)::mRFP::SL2::EGFP (M1)]*	
JN1730	*peEx1730[gpc-1p::daf-2 E11-E11.5(+c)-E12(-c)::mRFP::SL2::EGFP (M2)]*	
JN1731	*peEx1731[gpc-1p::daf-2 E11-E11.5(+c)-E12(-c)::mRFP::SL2::EGFP (M2)]*	
JN798	*peEx759[myo-3p::venus]*	Salt concentration learning assay
JN2702	*peEx1723[H20p::daf-2a, myo-3p::venus]*	
JN2703	*peEx1724[H20p::daf-2a, myo-3p::venus]*	
JN2704	*peEx1725[H20p::daf-2c, myo-3p::venus]*	
JN2705	*peEx1726[H20p::daf-2c, myo-3p::venus]*	
JN2706	*peIs2706[gcy-5p::daf-2c, unc-122p::mCherry]*	

JN1761	*peEx1761[asd-1p::venus]*	Expression analyses of splicing factors
JN1762	*peEx1762[fox-1p::venus]*	
JN1763	*peEx1763[asd-1p::spGFP1-10, fox-1p::spGFP1-10, H20p::spGFP11::mCherry]*	
JN1764	*peEx1764[ptb-1ap::spGFP1-10, ptb-1bp::spGFP1-10, asd-1p::spGFP11::mCherry, fox-1p::spGFP11::mCherry]*	
JN1765	*peEx1765[ptb-1ap::spGFP1-10, ptb-1bp::spGFP1-10, unc-75p::spGFP11::mCherry]*	
JN1766	*peEx1766[ptb-1ap::mCherry, ptb-1bp::mWasabi]*	
JN1767	*Ex[ptb-1ap::venus, ptb-1bp::venus, H20p::daf-2 E11-E11.5(+c)-E12::mRFP]*	
JN1768	*Ex[ptb-1ap::venus, ptb-1bp::venus, H20p::daf-2 E11-E11.5(+c)-E12(-c)::mRFP]*	

Supplementary Table 4. List of transgenic strains (mutant background)

Strain	Genotype	Used for
JN1769	*unc-75(e950); peIs785*	Expression analyses of the neuronal exon 11.5 skipping/inclusion reporter (the *peIs785* transgene)
JN1770	*unc-75(yb1714); peIs785*	
JN1771	*unc-75(yb1697); peIs785*	
JN1772	*unc-75(yb1700); peIs785*	
JN1773	*unc-75(yb1701); peIs785*	
JN1774	*unc-75(yb1723); peIs785*	
JN1775	*unc-75(yb1725); peIs785*	
JN1776	*hrpf-1(tm3406); peIs785*	
JN1777	*sym-2(gk150369); peIs785*	
JN1778	*ptb-1(gk113); peIs785*	
JN1779	*ptb-1(gk347274); peIs785*	
JN1780	*exc-7(ok370); peIs785*	
JN1781	*rsp-8(tm5638); peIs785*	
JN1782	*asd-1(yb1002); peIs785*	
JN1783	*asd-1(ok2299); peIs785*	
JN1784	*hrp-1(ok963) IV/nT1[qIs51] (IV;V); peIs785*	
JN1785	*Y59A8B.10(gk445934) ; peIs785*	
JN1786	*fox-1(e2643); peIs785*	
JN1787	*asd-1(ok2299); fox-1(e2643); peIs785*	
JN1788	*asd-1(ok2299)/qC1[qIs26] III; fox-1(e2643) X; peIs785*	
JN1789	*asd-1(yb1002); fox-1(e2643); peIs785*	
JN795	*unc-75(e950); ptb-1(gk113); peIs785*	
JN1790	*unc-75(e950); ptb-1(gk347274); peIs785*	
JN1793	*unc-75(e950); asd-1(ok2299); fox-1(e2643); peIs785*	
JN1794	*ptb-1(gk347274) II; asd-1(ok2299)/qC1[qIs26] III; fox-1(e2643) X; peIs785*	
JN1795	*unc-75(e950); peIs1709*	mRNA-tagging using the *peIs1709* transgene
JN1796	*ptb-1(gk347274); peIs1709*	
JN1797	*asd-1(ok2299); peIs1709*	
JN1798	*ptb-1(gk347274); asd-1(ok2299); peIs1709*	
JN1799	*asd-1(ok2299); peEx1707*	Analysis of *daf-2* exon 11.5 inclusion using the *peEx1707* transgene
JN2700	*fox-1(e2643); peEx1707*	
JN2701	*asd-1(ok2299); fox-1(e2643); peEx1707*	

JN1732	*ptb-1(gk347274); peIs785; peEx1732[casy-1p::ptb-1a::SL2::CFP, unc-122p::mCherry]*	Ectopic expression analysis of *ptb-1* cDNAs
JN1733	*ptb-1(gk347274); peIs785; peEx1733[casy-1p::ptb-1a::SL2::CFP, unc-122p::mCherry]*	
JN1734	*ptb-1(gk347274); peIs785; peEx1734[casy-1p::ptb-1b::SL2::CFP, unc-122p::mCherry]*	
JN1735	*ptb-1(gk347274); peIs785; peEx1735[casy-1p::ptb-1b::SL2::CFP, unc-122p::mCherry]*	
JN2707	*daf-2(pe1230); peEx1723*	Salt concentration learning assay
JN2708	*daf-2(pe1230); peEx1724*	
JN2709	*daf-2(pe1230); peEx1725*	
JN2710	*daf-2(pe1230); peEx1726*	
JN2711	*ptb-1(gk347274); asd-1(ok2299); peEx1723*	
JN2712	*ptb-1(gk347274); asd-1(ok2299); peEx1724*	
JN2713	*ptb-1(gk347274); asd-1(ok2299); peEx1725*	
JN2714	*ptb-1(gk347274); asd-1(ok2299); peEx1726*	
JN2715	*ptb-1(gk347274); asd-1(ok2299); peIs2706*	
JN2716	*daf-16(mgDf50); ptb-1(gk347274); asd-1(ok2299); peIs2706*	

Supplementary Table 5. Primer list

Primer name	Sequence (5'-3')	Target	Used for
E11_RTPCR Fw	CCGCAAATCGACAACACAC	*daf-2*	RT-PCR analysis of *daf-2* isoforms
E12_RTPCR Rv	TCCAATGCCAGAGCTGATAC		
E12 Fw	TGTATCAGCTCTGGCATTGG	*daf-2*	qRT-PCR analysis of *daf-2* isoforms
E13 Rv	AAGATGCTCCGGGTACTG		
E11/12 Fw	GTTTTAATGCCGAGAGACACG	*daf-2* *(exon 11.5-)*	
E12 Rv-1	ATCAGCGGCTTCTTTCCACC		
E11.5 Fw	CGATTGGACGGAAAGAATATGAACAG	*daf-2* *(exon 11.5+)*	
E12 Rv-2	TTTCCAACTCTTCACTGACTCG		
odr-3 Fw	CTGGCGTGAAGAAAGCATTTG	*odr-3*	Confirmation of neuron-type-specific poly(A) RNA isolation
odr-3 Rv	CCAGGTTCAGATATACGCTGAC		
glr-2 Fw	GGAGAGCCAGAATTGAAAGG	*glr-2*	
glr-2 Rv	AACGTCGTACTCGAATCCTG		
eef-1A.1 Fw	ATTGCCACACCGCTCACA	*eef-1A.1*	Internal standard for qRT-PCR analyses
eef-1A.1 Rv	CCGGTACGACGGTCAACCT		
ptb-1a Fw	ataggtaccATGACCAAGCGTGGCCCAGATGATTTGC	*ptb-1a*	Isolation of *ptb-1* cDNAs
ptb-1b Fw	ataggtaccATGGCAGCGCCACAAGTTCTCAC	*ptb-1b*	
ptb-1 Rv	CTAGATGCCGGATTTGGAGAAGGAAACACG	*ptb-1a* and *ptb-1b*	
Cbr-daf2 RT	GCTTGGCTCTCAAATAGTCG	*Cbr-daf-2*	Reverse transcription for identification of *Cbr-daf-2* isoforms
Cbr-daf2 Fw1	GACGAAATATGCGGTTCCAG		1st step PCR for identification of *Cbr-daf-2* isoforms
Cbr-daf2 Rv1	TCTTGGTCGCCGCTTTATG		
Cbr-daf2 Fw2	AGCGTTAGCATCCTCTGAC		2nd step PCR for identification of *Cbr-daf-2* isoforms
Cbr-daf2 Rv2	GCCAACATTTCATCCTGTTC		
ptb-1a_RTPCR Fw	GCAGCTGCGTTTGTCTCTGG	*ptb-1a* and *ptb-1b*	RT-PCR analysis of *ptb-1* isoforms
ptb-1b_RTPCR Fw	AGCATCCACAGGTCGAGCAG		
ptb-1a&b_RTPCR Rv	GTTGTGGCTCCGAATACTGG		
rpl-12_RTPCR Fw	CAAAGTTCGACCCAACTGAG	*rpl-12*	
rpl-12_RTPCR Rv	GCTGGGATCTCGATCTCTCC		

etr-1_RTPCR Fw	CCAGCTCAGCAAGAAGCACA	*etr-1*	
etr-1_RTPCR Rv	GTGAGGCTACTGCTTGATAG		

Supplementary References

1 Loria, P. M., Duke, A., Rand, J. B. & Hobert, O. Two neuronal, nuclear-localized RNA binding proteins involved in synaptic transmission. *Curr Biol* 13, 1317-1323 (2003).

2 Kuroyanagi, H., Watanabe, Y. & Hagiwara, M. CELF family RNA-binding protein UNC-75 regulates two sets of mutually exclusive exons of the *unc-32* gene in neuron-specific manners in *Caenorhabditis elegans*. *PLoS Genet* 9, e1003337, doi:10.1371/journal.pgen.1003337 (2013).

3 Barberan-Soler, S., Medina, P., Estella, J., Williams, J. & Zahler, A. M. Co-regulation of alternative splicing by diverse splicing factors in *Caenorhabditis elegans*. *Nucleic Acids Res* 39, 666-674, doi:10.1093/nar/gkq767 (2011).

4 Thompson, O. *et al.* The million mutation project: a new approach to genetics in *Caenorhabditis elegans*. *Genome Res* 23, 1749-1762, doi:10.1101/gr.157651.113 (2013).

5 Kuroyanagi, H., Kobayashi, T., Mitani, S. & Hagiwara, M. Transgenic alternative-splicing reporters reveal tissue-specific expression profiles and regulation mechanisms *in vivo*. *Nat Methods* 3, 909-915, doi:10.1038/nmeth944 (2006).

6 Kimura, K. D., Tissenbaum, H. A., Liu, Y. & Ruvkun, G. *daf-2*, an insulin receptor-like gene that regulates longevity and diapause in *Caenorhabditis elegans*. *Science* 277, 942-946 (1997).

7 Ohno, H. *et al.* Role of synaptic phosphatidylinositol 3-kinase in a behavioral learning response in *C. elegans*. *Science* 345, 313-317, doi:10.1126/science.1250709 (2014).

8 Skipper, M., Milne, C. A. & Hodgkin, J. Genetic and molecular analysis of *fox-1*, a numerator element involved in *Caenorhabditis elegans* primary sex determination. *Genetics* 151, 617-631 (1999).
